# Expanding Transition Metal-Mediated Bioorthogonal Decaging to Include C–C Bond Cleavage Reactions

**DOI:** 10.1021/jacs.3c01960

**Published:** 2023-05-03

**Authors:** Gean M. Dal Forno, Eloah Latocheski, Ana Beatriz Machado, Julie Becher, Lavinia Dunsmore, Albert L. St. John, Bruno L. Oliveira, Claudio D. Navo, Gonzalo Jiménez-Osés, Rita Fior, Josiel B. Domingos, Gonçalo J. L. Bernardes

**Affiliations:** † Department of Chemistry, Federal University of Santa Catarina—UFSC, Campus Trindade, Florianópolis, Santa Catarina 88040-900, Brazil; ‡ Champalimaud Centre for the Unknown, Champalimaud Foundation, Av. Brasilia, Lisboa 1400-038, Portugal; § Yusuf Hamied Department of Chemistry, University of Cambridge, Lensfield Road, Cambridge CB2 1EW, U.K.; ∥ Instituto de Medicina Molecular João Lobo Antunes, Faculdade de Medicina, Universidade de Lisboa, Av. Prof. Egas Moniz, Lisboa 1649-028, Portugal; ⊥ Center for Cooperative Research in Biosciences (CIC BioGUNE), Basque Research and Technology Alliance (BRTA), Bizkaia Technology Park, Building 800, Derio 48160, Spain; # Ikerbasque, Basque Foundation for Science, Bilbao 48013, Spain

## Abstract

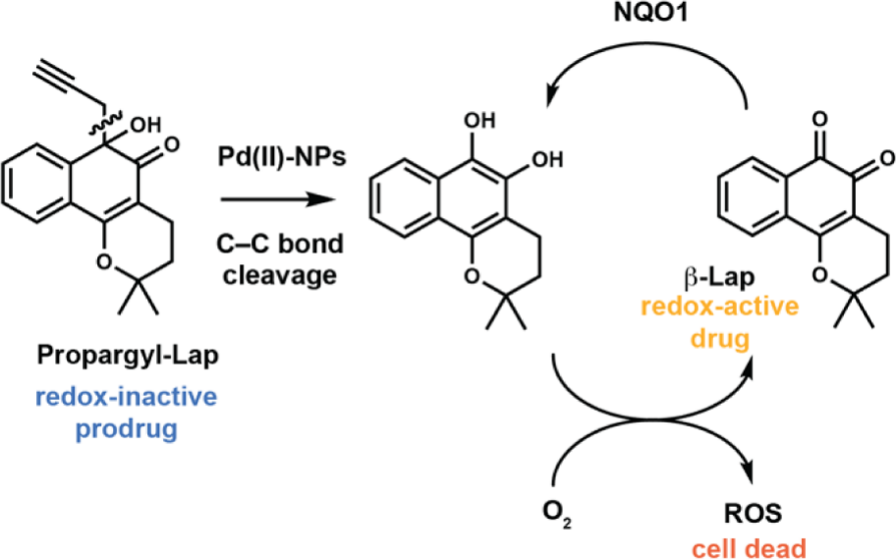

The ability to control the activation of prodrugs by transition metals has been shown to have great potential for controlled drug release in cancer cells. However, the strategies developed so far promote the cleavage of C–O or C–N bonds, which limits the scope of drugs to only those that present amino or hydroxyl groups. Here, we report the decaging of an *ortho*-quinone prodrug, a propargylated β-lapachone derivative, through a palladium-mediated C–C bond cleavage. The reaction’s kinetic and mechanistic behavior was studied under biological conditions along with computer modeling. The results indicate that palladium (II) is the active species for the depropargylation reaction, activating the triple bond for nucleophilic attack by a water molecule before the C–C bond cleavage takes place. Palladium iodide nanoparticles were found to efficiently trigger the C–C bond cleavage reaction under biocompatible conditions. In drug activation assays in cells, the protected analogue of β-lapachone was activated by nontoxic amounts of nanoparticles, which restored drug toxicity. The palladium-mediated *ortho*-quinone prodrug activation was further demonstrated in zebrafish tumor xenografts, which resulted in a significant anti-tumoral effect. This work expands the transition-metal-mediated bioorthogonal decaging toolbox to include cleavage of C–C bonds and payloads that were previously not accessible by conventional strategies.

## Introduction

Bioorthogonal cleavage of C–N and C–O bonds triggered by transition metals (TM) or small molecules enables conditional activation of small- and biomolecules in living systems.^1−3^ These reactions are now the basis of new cancer treatments in which a prodrug is activated preferentially at the tumor site. In 2020, the first bioorthogonal reaction entered Phase 1 clinical trials in humans for cancer therapy—a small-molecule-mediated inverse-electron-demand Diels–Alder decaging reaction.^4,5^

Bioorthogonal cleavage or decaging reactions mediated by TM are distinct from those triggered by small molecules because of the possibility of using sub-stoichiometric amounts of the catalytic metal to allow multiple reaction turnovers.^6^ The first example of the use of TM for bioorthogonal decaging was reported by Streu and Meggers in 2006 who used a Ru-mediated deallyloxycarbonyl (allyl carbamate) reaction in living cells for the activation of a fluorescent dye.^7^

**Figure 1:**
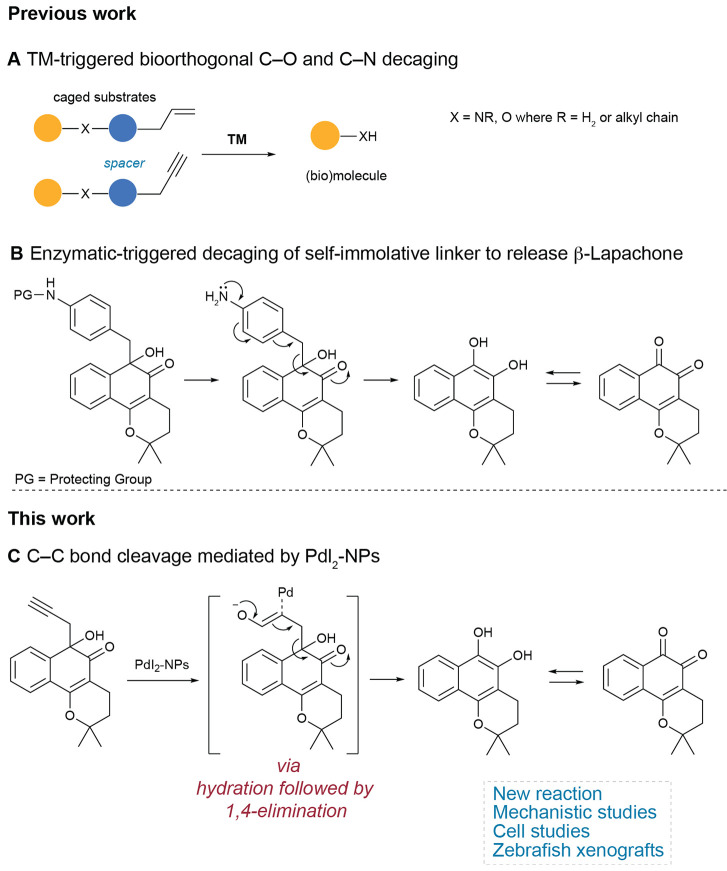
Palladium-mediated bioorthogonal bond cleavage strategies. (A) Palladium-mediated N/O-deallylation and depropargylation reactions for the activation of masked small molecules, proteins, and bioconjugates. (B) Specific enzymatic trigger, an acid-promoted, self-immolative C–C bond-cleaving 1,6-elimination mechanism releases β-lapachone.^30^ (C) This work, the decaging of the prodrug propargyl β-lapachone, through a palladium(II) nanoparticles-mediated C–C bond cleavage.

TM-mediated N/O-deallylation and depropargylation reactions are the most popular decaging reactions because of the chemoselectivity of the TM for the alkene/alkyne moieties. Upon complexation, subsequent TM activation of those moieties leads to nucleophilic attack, which in turn results in C–N or C–O bond cleavage and activation of the otherwise masked small molecules, proteins, and bioconjugates (Figure 1A). Although much less common, TM-mediated decaging of *O*-allenyl^8^ and pentynoyl tertiary amides^9^ of masked OH and NR_2_ substrates, respectively, has also been used to chemically rescue protein activity and activate prodrugs.

TM used to mediate bioorthogonal dissociation reactions include ruthenium,^10−13^ gold,^14−17^ copper,^18^ palladium,^19−25^ and more recently platinum.^9,26,27^ Of these, palladium in the form of complexes or nanoparticles has been the preferred choice due to its enhanced activity and relatively low toxicity. However, the requirements of rapid kinetics, biocompatibility and low toxicity, solubility, stability, and traceability of the metal source makes the use of metal complexes to mediate decaging reactions in living systems challenging. In contrast to complexes, palladium nanoparticles (Pd-NPs) can be rationally engineered to exhibit those properties, including the potential to limit catalysis intra-^22^ or extracellularly.^28,29^

To date, these bioorthogonal reactions have been limited to the decaging of OH and NH_2_ from protected alkylated amines, carbamates, or ethers. As such, the development of new reactions for the protection of other functionalities is desirable to expand conditional activation of other functional groups and molecules. Our group has recently demonstrated the activation of *ortho*-quinones in treating cancer—upon a specific enzymatic trigger, an acid-promoted, self-immolative C–C bond-cleaving 1,6-elimination mechanism releases the redox-active hydroquinone intracellularly (Figure 1B). This strategy was used to build an antibody–drug conjugate of protected *ortho*-quinone β-lapachone (β-Lap), which led to targeted antitumor activity in a xenograft murine model of acute myeloid leukemia.^30^ β-Lap (**1**) is a natural *ortho*-quinone obtained from the bark of the Brazilian Lapacho tree with potential use in cancer therapy but, like other *ortho*-quinones, has limited application due to its systemic side effects. β-Lap has antitumor effects in a broad spectrum of cancer cells with high expression of NQO1, including breast,^31^ non-small-cell lung,^32^ pancreatic,^32^ prostate,^33^ colorectal,^34^ liver,^35^ and ovarian.^36^ In these cancers, β-Lap undergoes a redox cycle, which results in the formation of reactive oxygen species (ROS) and subsequent cell death.^37,38^ NQO1 reduces β-Lap to its hydroquinone form that undergoes a two-step spontaneous oxidation process to reform β-Lap, perpetuating the redox cycle. As such, strategies to mask and reveal the activity of β-Lap, as well as other *ortho*-quinones, are sought after.

In this study, we aimed to expand the toolbox of available bioorthogonal dissociative reactions by investigating a new C–C bond cleavage catalyzed by palladium for the activation of *ortho*-quinones. We demonstrate that a *C*-propargyl attached to the *ortho*-quinone carbon can be successfully decaged in an aqueous buffer and living cells using nontoxic amounts of palladium (II) nanoparticles (Figure 1C). This strategy was successfully applied to deliver β-Lap in zebrafish tumor xenografts. While preparing this manuscript, Zhang and co-workers reported the release of β-Lap through a C–C bond-cleaving elimination reaction which, in their case, took place upon deprotection of a boronic acid (ester) group by ROS.^39^

## Results and Discussion

### Design and Synthesis of β-Lap Prodrugs for Palladium-Mediated C–C Decaging Reaction

**Figure 2:**
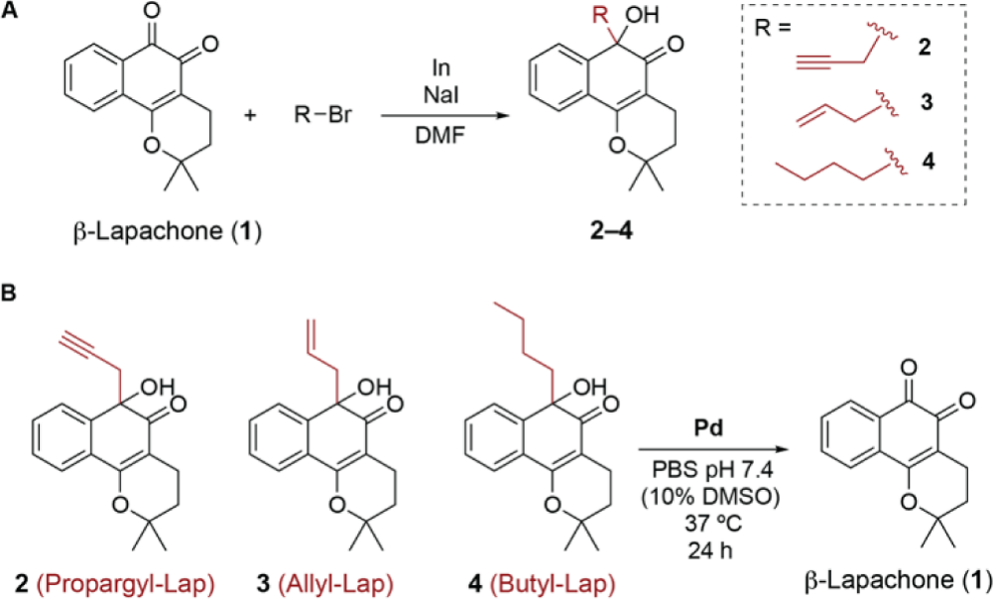
(A) Synthesis of β-Lap prodrugs (**2**–**4**) and (B) palladium-mediated decaging reaction.

Taking into consideration previous studies that showed TM decaging reactions from unsaturated groups and the cleavage of a C–C bond to release *ortho*-quinones through a self-immolative 1,6-elimination mechanism,^30^ we hypothesized that unsaturated protected derivatives of β-Lap could potentially be decaged by palladium-mediated C–C bond cleavage under biocompatible conditions. For masking of the *ortho*-quinone β-Lap, we selected propargyl and allyl protecting groups and the palladium non-sensitive group *n*-butyl as a negative control to confirm that the decaging reaction occurs through C–C bond cleavage and not by interaction with the other functional groups of β-Lap. The synthesis of β-Lap prodrugs was performed using a method adapted from the literature,^30,40^ and indium-mediated reductive alkylation of 1,2-diones was used (Figure 2A). The reaction takes place between β-Lap and different bromides in the presence of metallic indium and sodium iodide in dimethylformamide at room temperature and is aided by sonication. The reaction proved to be selective in the formation of α-hydroxyketones in good yields (see the Supporting Information). Since it has been shown that α-hydroxyketones do not display the redox cycling activity described for β-Lap,^30^ which is the main factor affecting its cell toxicity,^41^ the new molecules derived from β-Lap may be used as prodrugs, with toxicity restored upon the triggering of the decaging reaction.

### Palladium-Mediated C–C Decaging under Biocompatible Conditions

The ability of palladium to mediate the C–C bond cleavage of the prodrugs was evaluated under conditions that resemble a biological environment. Reactions were carried out by incubating 50 μM of prodrug substrates (**2**–**4**) in PBS pH 7.4/10% dimethyl sulfoxide (DMSO) for 24 h at 37 °C with palladium sources (Figure 2B, Table 1). Reactions were monitored by liquid chromatography–mass spectrometry (LC–MS) with the observation of the β-Lap *m/z* signal ([M + H]^+^, *m/z* 243, retention time, 11.5 min). To determine the yields, a calibration curve was constructed using product standards of known β-Lap amounts (Figure S18, Supporting Information).

**Table 1:** Reaction Analysis of the Palladium-Mediated C–C Decaging Reactions of **2**–**4** by LC–MS^a^

entry	Pd source	protecting group	yield (%)^b^
1	—	Propargyl (**2**)	0
2	—	Allyl (**3**)	0
3	Na_2_PdCl_4_	*n*-Butyl (**4**)	0
4	Na_2_PdCl_4_	Propargyl (**2**)	21
5	Na_2_PdCl_4_	Allyl (**3**)	13
6	PdI_2_-NPs	Propargyl (**2**)	66
7	PdI_2_-NPs	Allyl (**3**)	31
8	Pd(0)-NPs	Propargyl (**2**)	4
9	Pd(0)-NPs	Allyl (**3**)	0
10	PdI_2_	Propargyl (**2**)	0
11	PdI_2_ + PVP^c^	Propargyl (**2**)	0
12	PdI_2_-NPs + EDTA^d^	Propargyl (**2**)	8

a 50 μM of the substrate and equimolar amounts of the Pd source.

b Determined by LC–MS.

c 5 equivalents of PVP.

d 10 equivalents of EDTA.

Initially, the stability of the new substrates was evaluated in the absence of any type of trigger (entries 1 and 2, Table 1). As expected, spontaneous β-Lap formation was not observed, as the C–C bond is very strong. This contrasts with other protecting groups, *e.g.*, carbonates and carbamates, which may spontaneously cleave under biological conditions.^42^ The stock solutions in DMSO (4 mM) were also stable for several months on the bench, demonstrating the effectiveness of substrate protection. Then the decaging reaction was performed with different palladium sources, which included Na_2_PdCl_4_ salt as well as Pd-NPs. When compounds **2** (propargyl) and **3** (allyl) were reacted with an equimolar amount of Na_2_PdCl_4_ salt, we observed C–C cleavage, although in low yields, 21 and 13%, respectively (entries 4 and 5, Table 1). When compound **4** (*n*-butyl) was mixed with a large excess of Na_2_PdCl_4_ (5.0 equivalents), no β-Lap formation was detected (entry 3, Table 1), which suggests that the decaging reaction indeed occurs between the metal and the unsaturated protecting groups of compounds **2** and **3**. These results showed that our hypothesis was feasible and encouraged us to pursue the search of optimal conditions for C–C bond cleavage in this unprecedented palladium-mediated *ortho*-quinone deprotection reaction.

Although palladium complexes can mediate deprotection reactions under biological conditions, cell and *in vivo* applications have been most successful using Pd-NPs.^43,44^ The reasons for choosing Pd-NPs include their low toxicity, the possibility of targeting, and local administration to enable tissue-specific activation (*e.g*., in a tumor) and thus reduce potential side toxicity.^28,29^

For this work, we selected palladium(II) iodide (PdI_2_-NPs) and palladium(0) [Pd(0)-NPs] nanoparticles, both with similar size (2 nm), varying only in the oxidation state of palladium. The PdI_2_-NPs were previously demonstrated to be efficient catalysts in C–C coupling reactions in water^45^ and decaging of prodrugs in living cells in C–O cleavage reactions.^46^ The most efficient C–C bond cleavage occurred for the pair PdI_2_-NPs and Propargyl-Lap (**2**) with a 66% yield (entry 6, Table 1). Allyl-Lap (**3**) was less reactive, affording a 31% yield (entry 7, Table 1). Interestingly, Pd(0)-NPs proved to be almost unreactive in promoting C–C bond cleavage for both substrates (entries 8 and 9, Table 1), which suggests that Pd(II) is the active palladium catalytic species.

Following these results, we decided to compare our PdI_2_-NPs with commercially available PdI_2_ powder in the presence or absence of polyvinylpyrrolidone (PVP, a stabilizing agent). PdI_2_, both with and without PVP, did not show C–C bond cleaving activity under identical conditions to those used for PdI_2_-NPs (entries 10 and 11, Table 1). This result suggests that the nanoparticulate form of the PdI_2_-NPs is responsible for their activity relative to commercially available PdI_2_. Additionally, the addition of EDTA (10 equivalents) to the reaction medium abolishes reactivity from 66 to only 8% (entry 12, Table 1). EDTA is a chelating agent that readily forms a strong stable complex with Pd(II), which inhibits substrate coordination to Pd and consequently catalysis. This poisoning experiment supports the participation of Pd(II) as the active species in the C–C depropargylation reaction of the Propargyl-Lap prodrug. The full set of control experiments including the effects of buffer and Pd concentration is shown in Table S1 (Supporting Information).

The Pd(II)-mediated cleavage of Propargyl-Lap (**2**) and Allyl-Lap (**3**) (40 μM) at 37 °C in water was also investigated by electrospray ionization mass spectrometry in positive mode [ESI–MS(+)]. ESI–MS may be used for the identification of ions in solution and complex reaction mixtures. Four aliquots were collected from the reaction vessel at different reaction times: at 0 (just after mixing the reagents), 15, 30, and 90 min of reaction (Figures S22–S29, Supporting Information). Initially, several clusters for β-Lap were detected in the reaction of **2**, but no Pd-intermediates, which suggests a relatively fast depropargylation step. Indeed, substrate **2** practically disappears after just 10 min, while the allyl substrate **3** is observed in a reasonable amount even after 90 min. A fast depropargylation is also observed for C–N and C–O bond cleavage reactions, which indicates that a similar metal binding mode and cleavage mechanism may be operating.^43^ These observations agree well with our theoretical calculations shown next.

### Mechanistic Studies of the Palladium-Mediated C–C Bond Cleavage

We performed a computational study to help understand the mechanism of the C–C bond cleavage of protected β-Lap substrates (**2**–**4**), either spontaneously or mediated by Pd(II) species. First, we evaluated the possibility of directly breaking the C–C bond (highlighted in red, Figure S30, Supporting Information) by analyzing the potential energy surface (PES) along that bond. As with the *para*-aminobenzyl linker (PAB-Lap),^30^ no energy maximum (*i.e*., transition state, TS) was detected for any of the three analyzed systems.

Similar profiles were obtained for the carbonyl-protonated species (highlighted in blue, Figure S31, Supporting Information) indicating that, unlike PAB-Lap derivatives,^30^ these protonated derivatives remain unreactive in solution, requiring an additional driving force. We then considered that the Pd(II) catalyst may act as a Lewis acid coordinating to both oxygen atoms of the quinone moiety to promote the elimination reaction. However, the same profile was obtained when scanning the cleavage of the C–C bond, which suggests no reactivity (Figure S32, Supporting Information). Together these observations point toward a different Pd-promoted mechanism.

**Figure 3:**
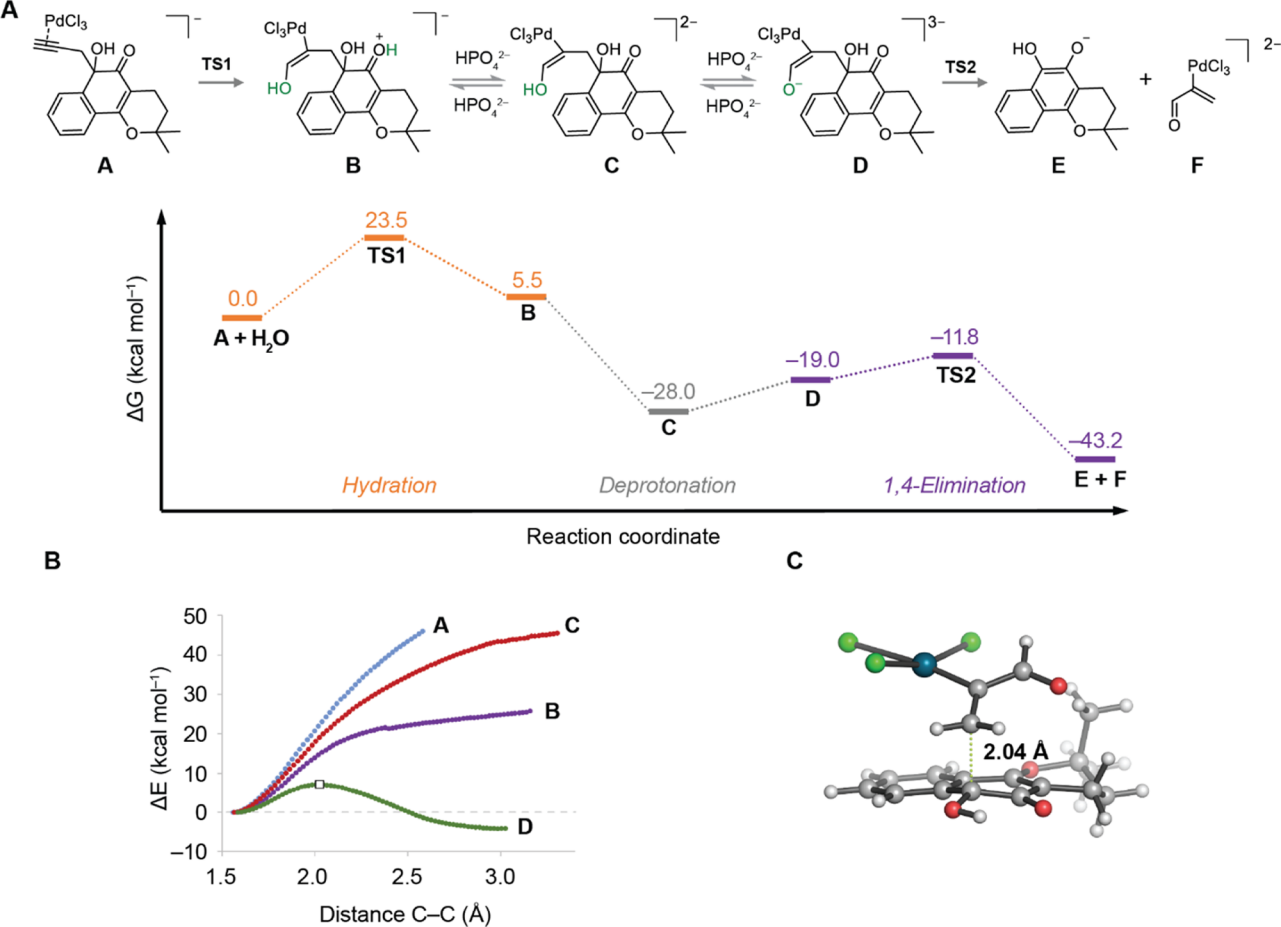
(A) Minimum energy profile (kcal mol^–1^) calculated with PCM(H_2_O)/ωB97x-D/6-311+G(2d,p) +LanL2DZ(Pd)//PCM(H_2_O)/M06-2X/6-31+G(d,p)+LanL2DZ(Pd) for the first turnover of the depropargylation reaction catalyzed by [PdCl_4_]^2–^ in water. (B) PES calculated at the same theory level for the elimination of intermediates **A** (blue), **B** (purple), **C** (red), and **D** (green). All the scans were started from the lowest energy ground state conformer for each derivative. Only the PES for **D** displayed a maximum corresponding to the elimination reaction (white square), whereas no maximum was detected for the other intermediates. (C) Geometry of the lowest-energy calculated decaging transition state (**TS2**) Interatomic distances are given in angstroms. C–C breaking bonds are shown as dotted green lines.

Next, we envisioned that Propargyl-Lap **2** may undergo a similar reaction mechanism as the one proposed by Coelho *et al.*(47) for the depropargylation of ether derivatives (Figure 3A). The proposed mechanism involves an anti-Markovnikov attack of a water molecule (**TS1**) at the terminal position of the propargyl Pd-complex **A** (hydration), which is the rate-limiting step (Δ*G*^‡^ = 23.5 kcal mol^–1^) of the whole process, to form the corresponding oxonium intermediate (**B**). Notably, one of the hydrogen atoms of the attacking water molecule is concomitantly transferred to the carbonyl group of the β-Lap. Intermediate **B**, containing an activated (*i.e.*, protonated) carbonyl group and a neutral enol group, might undergo elimination similar to that of PAB-Lap.^30^ However, no transition structure for the elimination reaction was detected upon analysis of the C–C bond breaking PES (Figure 3B), as a result of the lower nucleophilicity of the enol group in comparison to the aniline group present in PAB-Lap.^30^ Similarly, enol intermediate **C**, formed upon deprotonation of **B**, also showed an uphill profile on the C–C bond breaking PES, as expected for the reduced nucleophilicity and electrophilicity of the neutral enol and carbonyl groups, respectively (Figure 3B). A second deprotonation by the buffer to yield enolate **D** is required to promote the fast 1,4-elimination reaction releasing the β-Lap upon protonation and oxidation. Therefore, a fully-developed negative charge at the enol *O*-atom after water addition is mandatory for the 1,4-elimination to take place (Figure 3B), resembling some reaction pathways calculated previously for PAB-Lap (the NH^–^ species was very reactive toward 1,6-elimination).^30^ The **TS2** (Δ*G*^‡^ = 9.0 kcal mol^–1^) for C–C bond breaking is shown in Figure 3C.

On the other hand, hydration of Allyl-Lap (**3**) leads to a dead-end pathway since the hydrated product has no double bond adjacent to a deprotonated *O-*atom and, hence, cannot undergo elimination. However, Allyl-Lap may react with Pd(0) species [either added as the catalyst or formed under the reaction conditions (Wacker reaction)], in a similar way to the well-known deprotection reaction of allyl ether/carbamates (by a Tsuji–Trost-type reaction).^43^ Finally, the lack of unsaturated bonds on the Butyl-Lap (**4**) prevents Pd coordination and, hence, hydration and subsequent elimination.

A possible mechanism involving an initial Markovnikov attack of the water molecule at the C2 position of the propargyl moiety was also evaluated. However, this pathway has a very high calculated activation barrier (nearly 31 kcal mol^–1^) for the C–C cleavage step and was therefore discarded.

### Palladium(II)-Nanoparticles Mediated C–C Bond Cleavage in Living Cells

**Figure 4:**
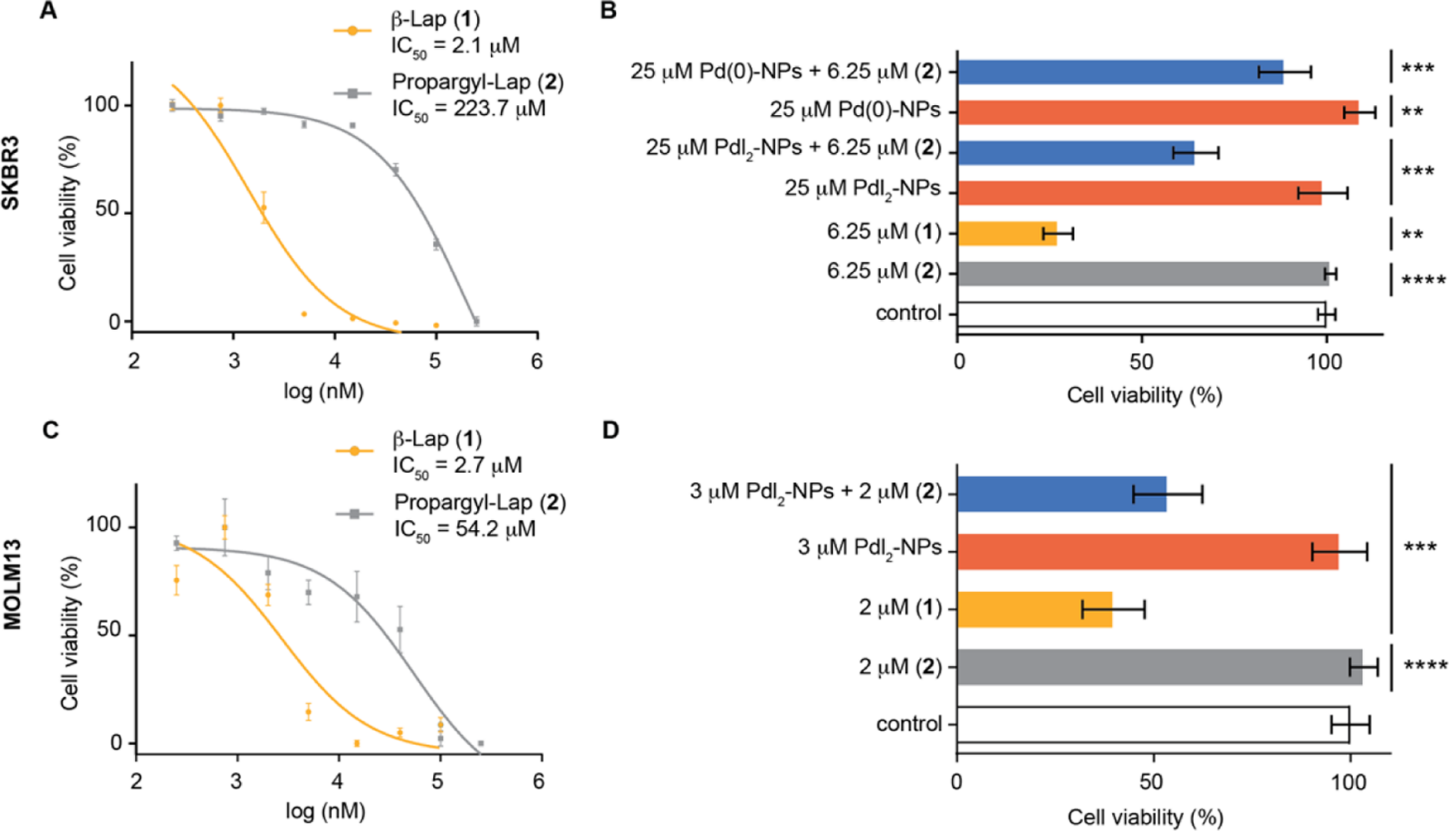
Toxicity of Propargyl-Lap (**2**) compared to β-lapachone for cancer cell lines measured by CellTiter Blue assay. (A) SKBR3 cell viability for 72 h. (B) Cell viability of SKBR3 cells after treatment with Propargyl-Lap (**2**) and subsequent decaging efficiency upon treatment with Pd(0)-NPs or PdI_2_-NPs, after 72 h (C) MOLM13 cell viability for 48 h. (D) Cell viability of MOLM13 cells after treatment with Propargyl-Lap (**2**) and subsequent decaging efficiency upon treatment with PdI_2_-NPs, after 48 h. Cell viability was determined by CellTiter Blue assay. The statistical significance: **P ≤ 0.01, ***P ≤ 0.001, and ****P ≤ 0.0001. Each experiment was performed in technical triplicates and two biological experiments. Error bars represent the standard error of the mean. Cells were treated with increasing concentrations of **2** and β-Lap during 48 h, after which the cell viability was determined using the CellTiter Blue assay. IC_50_ values were calculated from the generated eight-point semilog dose–response curves.

For living cell activation studies, we chose Propargyl-Lap (**2**) as the substrate, since it showed faster decaging in aqueous solution. First, we determined the toxicity of **2** and β-Lap (**1**) in human breast cancer (SKBR3) and acute monocytic leukemia (MOLM13) cells. Propargyl-Lap displayed a significant reduction in cytotoxicity relative to β-Lap (approx. 107- and 20-fold in SKBR3 and MOLM13, respectively, Figure 4A,C), which demonstrates the suitability of the propargylic group to mask the redox-cycling activity of the *ortho*-quinone. Next, we showed that the prodrug (**2**) is stable in the growth media used to culture SKBR3 (McCoy’s medium) and MOLM13 (RPMI medium), respectively (Figures S34, S35, Supporting Information). To test palladium-mediated cleavage of C–C bonds in *ortho*-quinones, we chose non-toxic Pd-NPs with oxidation states II (PdI_2_-NPs) or 0 [Pd(0)-NPs].^45,46^ Unmasking of (**2**) to generate toxic β-Lap was determined by incubating the cells with PdI_2_-NPs, Pd(0)-NPs, or Propargyl-Lap alone (negative controls), β-Lap (positive control), and Propargyl-Lap + NPs (reaction). Viability was assessed using CellTiter Blue. In brief, SKBR3 cells were incubated for 72 h with 6.25 μM Propargyl-Lap (**2**) and 25 μM Pd-NPs, whereas MOLM13 cells were incubated for 48 h with 2 μM Propargyl-Lap (**2**) and 3 μM Pd-NPs. The concentrations chosen were those at which no Pd-NPs toxicity was detected (Figures S36 and S37, Supporting Information). We found that when **2** was incubated in the presence of PdI_2_-NPs, a significant decrease in cell viability was observed as a result of the formation of active β-Lap in both cell lines (Figure 4B,D). Importantly, when using Pd(0)-NPs, very little toxicity was detected, which corroborates our finding that Pd(0) is not able to catalyze the C–C bond cleavage. Additionally, when Na_2_PdCl_4_ was used as the reaction promoter, a significant decrease in the efficiency of the decaging reaction and, therefore, in restoring the toxicity of β-Lap was observed for both cell lines (Figure S38, Supporting Information).

### Palladium(II)-Nanoparticles Mediated C–C Decaging *In Vivo*

To test the *in vivo* unmasking of Propargyl-Lap (**2**) by PdI_2_-NPs, we used a zebrafish larvae xenograft model.^48^ This model is a fast *in vivo* platform with a resolution that allows analyses of crucial hallmarks of cancer including metastatic and angiogenic potentials while also allowing discrimination of differential anticancer therapy responses at single-cell resolution.^49−52^ We started by assessing the maximum tolerated concentration of each compound, alone and in combination, in non-injected zebrafish larvae (Figure S39, Supporting Information). Propargyl-Lap (**2**) was diluted in the E3 medium, and PdI_2_-NPs were either injected into the perivitelline space (PVS) or also diluted in E3. According to the observed toxicity, the concentrations that were chosen for the following assays were 5 μM of Propargyl-Lap (**2**), diluted in the embryo medium, and 5 μM of PdI_2_-NPs injected into the PVS.

**Figure 5:**
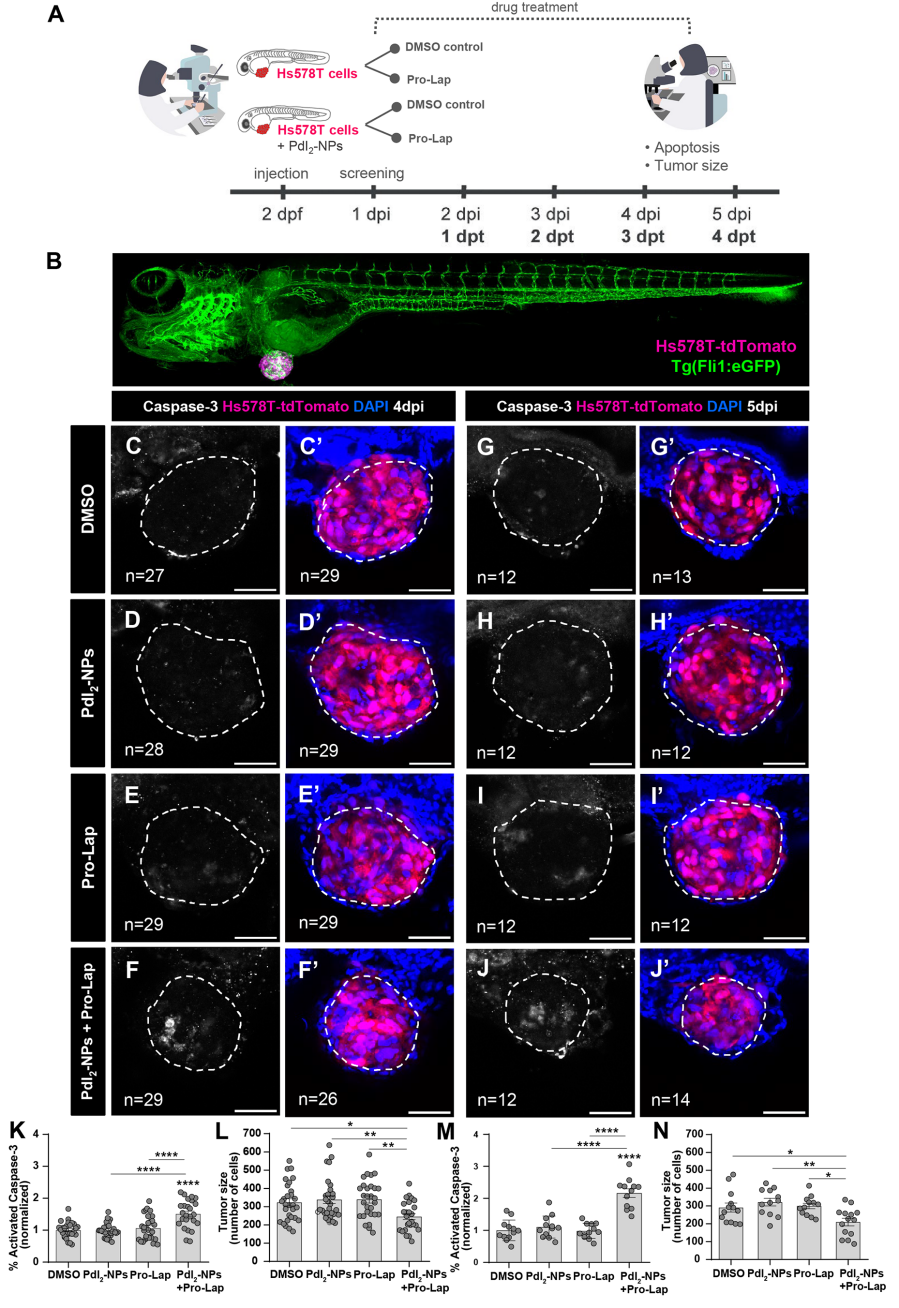
PdI_2_-NPs-mediated Propargyl-Lap decaging in zebrafish xenografts. (A) Experimental design: Hs578T-tdTomato TNBC cells were injected either alone or together with the PdI_2_-NPs (5 μM) into the PVS of 2 days post fertilization zebrafish embryos. At 24 hpi, xenografts were randomly distributed into two treatment groups: DMSO (control) and Propargyl-Lap, with daily E3/drug renewal. At 4 and 5 dpi, corresponding to 3 dpt and 4 dpt, xenografts were fixed and analyzed for apoptosis and the tumor size was quantified. (B) Representative low magnification of a Hs578T zebrafish xenograft injected in a Tg(Fli:eGFP) background. At 4 dpi (C–F’) and 5 dpi (G–J’), the xenografts were fixed, subjected to immunofluorescence, and later imaged by confocal microscopy (DAPI in blue, Hs578T-tdTomato in pink, and activated caspase-3 in white). Apoptosis [activated caspase 3, fold induction normalized to DMSO controls: (K,M); DMSO vs PdI_2_-NPs + Pro-Lap, 4 dpi (C *vs* F) ****P < 0.0001, 5 dpi (G *vs* J) ****P < 0.0001; PdI_2_-NPs vs PdI_2_-NPs + Pro-Lap, 4 dpi (D *vs* F) ****P < 0.0001, 5 dpi (H *vs* J) ****P < 0.0001; Propargyl-Lap vs PdI_2_-NPs + Propargyl-Lap, 4 dpi (E *vs* F) ****P < 0.0001, 5 dpi (I *vs* J) ****P < 0.0001] and tumor size [n° of tumor cells: (L,N); DMSO vs PdI_2_-NPs + Pro-Lap, 4 dpi (C’ *vs* F’) *P = 0.0274, 5 dpi (G’ *vs* J’) *P = 0.0407; PdI_2_-NPs vs PdI_2_-NPs + Pro-Lap, 4 dpi (D’ *vs* F’) **P = 0.0057, 5 dpi (H’ *vs* J’) **P = 0.003; Propargyl-Lap vs PdI_2_-NPs + Propargyl-Lap, 4 dpi (E’ *vs* F’) **P = 0.0049, 5 dpi (I’ *vs* J’) *P = 0.0222] were analyzed and quantified. Graphs are presented as average ± standard error of the mean. Results are from two independent experiments at 4 dpi and from one experiment at 5 dpi. The number of xenografts analyzed is indicated in the representative images, and each dot in the graphs represents one zebrafish xenograft. Statistical analysis was performed using an ANOVA test. Statistical results: ns > 0.05, *P ≤ 0.05, **P ≤ 0.01, ***P ≤ 0.001, and ****P ≤ 0.0001. All images are anterior to the left, posterior to right, dorsal up, and ventral down. Scale bar: 50 μm. PdI_2_-NPs—palladium (II) iodide nanoparticles; Pro-Lap—Propargyl-Lap.

Next, triple-negative breast cancer (TNBC) Hs578T zebrafish xenografts were generated as described previously.^48,49^ TNBC cells were injected either alone or together with the PdI_2_-NPs (5 μM) into the PVS of 2 days post-fertilization zebrafish embryos. At 24 h post-injection (hpi), xenografts (tumor cells only or tumor cells + PdI_2_-NPs) were randomly distributed into two treatment groups: DMSO (control) and Propargyl-Lap (5 μM), which were diluted in the E3 medium and renewed daily. At 4 and 5 days post-injection (dpi), corresponding to 3 and 4 days post-treatment (dpt), xenografts were fixed and subjected to immunofluorescence for activated caspase-3 (apoptosis). The extent of uncaging of the prodrug (**2**) by PdI_2_-NPs was analyzed through the quantification of induction of apoptosis and tumor size reduction, using confocal microscopy (Figure 5A–N).

In the single treatment groups with either PdI_2_-NPs or Propargyl-Lap, no significant induction of apoptosis (Figure 5L,N) nor reduction of tumor size (Figure 5K,M) were observed relative to DMSO control, which supports the low toxicity of both compounds tested in non-tumor-bearing zebrafish. In contrast, the combinatorial treatment of PdI_2_-NPs + Propargyl-Lap induced a significant anti-tumoral effect, manifested by an ∼1.5-fold increase in apoptosis at 4 dpi (****P < 0.0001) and an ∼2.2-fold increase at 5 dpi (****P < 0.0001), relative to both the DMSO control and the single treatments (Figure 5L,N). This increase in apoptosis was reflected in a reduction of ∼23–42% in tumor size in the combinatorial treatment condition relative to the single treatments, in both time points (Figure 5K,M). As a positive control, Hs578T xenografts were also subjected to treatment with β-Lap (**1**) alone, which showed a similar ∼1.6-fold increase in apoptosis at 4 dpi (***P = 0.0001), relative to the DMSO control (Figure S40). Finally, analysis of apoptosis in the tails of control and treated zebrafish by activated-caspase 3 staining showed no cell death in non-tumor tissues (Figure S41). This result is significant since the tails of zebrafish contain neuromasts (indicated by the white arrows in Figure S41), which are small sensory organs known to be particularly prone to cell death.

Our *in vivo* results suggest a successful drug decaging mediated by PdI_2_-NPs, which induces tumor cell death by apoptosis. This work constitutes the first demonstration of metal-mediated bioorthogonal cleavage of a C–C bond and its utility for drug activation *in vivo*.

## Conclusions

In summary, a novel C–C cleavage reaction catalyzed by palladium(II) nanoparticles for the release of *ortho*-quinones (β-Lap) from otherwise stable α-hydroxyketones prodrugs, both in mammalian cell culture and in living organisms, has been reported. The C-propargyl (**2**) derivative could be decaged through a palladium (II)-mediated anti-Markovnikov hydration of the alkyne moiety and 1,4-elimination reaction, releasing the free *ortho*-quinone upon protonation and oxidation. Application of non-toxic Pd(II)-mediated depropargylation in cells led to unmasking and release of the active drug. Importantly, this C–C bond cleavage reaction took place *in vivo* and was applied to by demand release of β-Lap in a zebrafish xenograft model of cancer. This work expands the toolbox of available TM-mediated bioorthogonal decaging reaction to include for the first time C–C bond cleavage reactions.

## Supporting Information Available

Detailed methods and characterization data (PDF)
