# Supplementary material for: Expanding Transition Metal-Mediated Bioorthogonal Decaging to Include C–C Bond Cleavage Reactions
Source: J Am Chem Soc. 2023 May 3;145(19):10790–9. doi: 10.1021/jacs.3c01960 (PMC10197128; doi:10.1021/jacs.3c01960)
Supplement: Supplementary file 1 — ja3c01960_si_001.pdf [file ja3c01960_si_001.pdf]

Supporting Information

Expanding Transition Metal-Mediated Bioorthogonal Decaging to Include C–C Bond Cleavage Reactions

Gean M. Dal Forno,[1] Eloah Latocheski,[1] Ana Beatriz Machado,[2] Julie Becher,[3] Lavinia Dunsmore,[3] Albert L. St. John,[1] Bruno L. Oliveira,[4] Claudio D. Navo,[5] Gonzalo Jiménez-Osés,[5,6] Rita Fior,[2] Josiel B. Domingos[1*] and Gonçalo J. L. Bernardes[3,4*]

1 Department of Chemistry, Federal University of Santa Catarina - UFSC, Campus Trindade, Florianópolis - SC, 88040-900 (Brazil)

2 Champalimaud Centre for the Unknown, Champalimaud Foundation, Av. Brasilia, 1400-038 Lisboa (Portugal)

3 Yusuf Hamied Department of Chemistry, University of Cambridge, Lensfield Road, Cambridge CB2 1EW (UK)

4 Instituto de Medicina Molecular João Lobo Antunes, Faculdade de Medicina, Universidade de Lisboa, Av. Prof. Egas Moniz, 1649-028 Lisboa (Portugal)

5 Center for Cooperative Research in Biosciences (CIC BioGUNE), Basque Research and Technology Alliance (BRTA), Bizkaia Technology Park, Building 800, 48160 Derio (Spain)

6 Ikerbasque, Basque Foundation for Science, 48013 Bilbao (Spain)

* Correspondence should be addressed to G.J.L.B. and J.B.D.:
E-mail: *gb453@cam.ac.uk* and *josiel.domingos@ufsc.br*

Table of Contents

1. Materials and Methods 3

1.1. General Information 3

1.2. Quantum Mechanical calculations 3

1.3. Breast Cancer Zebrafish Xenograft Experiments 4

2. Synthetic Procedures and Compounds Characterization 6

3. Reaction analysis of the palladium-mediated C–C decaging reaction of compounds 2-4 by LC-MS 18

4. LC-MS analysis for the C–C decaging of Propargyl-Lap mediated by PdI_2-NPs 20

5. ESI-MS(+) for the C–C decaging of Propargyl-Lap mediated by Na_2PdCl_4 in water 21

6. ESI-MS(+) for the C–C decaging of Allyl-Lap mediated by Na_2PdCl_4 in water 23

7. Quantum Mechanical data 25

8. Prodrug stability in cell medium 32

9. Cell viability assays 33

10. Propargyl-Lap (2) C–C Decaging in Cancer Cells: Na_2PdCl_4 35

11. Maximum Tolerated Concentration assay in zebrafish larvae 36

12. References 38

1. Materials and Methods

1.1. General Information

Solvents, Reagents and Materials: All the reagents and solvents were purchased from commercial suppliers and used as received. DMF was stored with 4 Å molecular sieves. Ultrapure water (resistivity of 18.2 Ωm) was used in preparation of buffers (Phosphate Buffer and PBS). Water for HPLC or LC-MS was HPLC grade and H_2O for synthesis was deionized. Column chromatography was performed on silica gel (70–230 mesh). Monitoring of reactions was performed using TLC Silica gel 60 F254 plates. Compounds were detected using shortwave (254 nm) UV lamp.

Characterization: NMR ^{1}H and ^{13}C spectra were acquired on a Varian FT-NMR 200 MHz 400 (^{1}H at 200 MHz and ^{13}C at 50 MHz) or BRUKER AVANCE III HD 700 (^{1}H at 700 MHz and ^{13}C at 175 MHz at 25 °C, using $CDCl_3$ as the solvent and tetramethylsilane (TMS) as the internal standard. Melting points were carried out on a Stanford Research Systems OptiMelt and are uncorrected. High resolution mass spectra were obtained with a Waters Xevo GS-2 QTof UPLC-MS and ionized by electrospray (ESI).

HPLC-MS: The yield of C-C bond cleavage was determined using a Shimadzu Prominence fitted with LCMS-2020 (column temp 25 °C, UV = 190 – 600 nm, MS - ESI) at a flow rate of 0.15 mL/min using a solvent system of 70% A + 30% B for 20 min (A = 0.1% formic acid in H_2O, B = MeCN). The column used was a Shim-pack XR-ODS (2,2 µm, 30 mm, 2 mm i.d.).

ESI-MS: The electrospray ionization-mass spectrometry (ESI-MS) experiments were performed in an Amazon X Ion Trap mass spectrometer (Bruker Daltonics). Aliquots of the reaction medium were periodically taken, diluted in water and eluted with a flow rate of 5.0 µL min^{-1}. The samples were analyzed in the positive ion mode. ESI parameters were as follows: Trap Drive 48.7, Capillary Exit 140.0 V, Dry Temp 180 °C, Nebulizer 10.15 psi, Dry Gas 4.00 l/min, HV Capillary 4500 V, HV End Plate Offset -500 V. The mass spectra acquisition range was 50–2800 m/z.

1.2. Quantum Mechanical calculations

Full geometry optimizations and transition structure (TS) searches were carried out with Gaussian 16[1] using the M06-2X hybrid functional,[2] 6-31+G(d,p) basis set and LanL2DZ[3] effective core potential for palladium atoms with ultrafine integration grids. Bulk solvent effects in water were considered implicitly through the IEF-PCM polarizable continuum model.[4] The possibility of different conformations was considered for all structures. All stationary points were characterized by a frequency analysis performed at the same level used in the geometry optimizations from which thermal corrections were obtained at 298.15 K. Single point calculations were performed on those geometries using the ωB97x-D hybrid functional,[5] 6-311+G(2d,p) basis set and LanL2DZ effective core

potential for palladium atoms with ultrafine integration grids. The quasiharmonic approximation reported by Truhlar et al. was used to replace the harmonic oscillator approximation for the calculation of the vibrational contribution to entropy.[6] Scaled frequencies were not considered. Mass-weighted intrinsic reaction coordinate (IRC) calculations were carried out by using the Hratchian and Schlegel algorithm[7] to ensure that the TSs indeed connected the appropriate reactants and products. Gibbs free energies (ΔG) were used for the discussion on the relative stabilities of the considered structures. The lowest energy conformer for each calculated stationary point was considered in the discussion; all the computed structures can be obtained from authors upon request. Electronic energies, entropies, enthalpies, Gibbs free energies, and lowest frequencies of the lowest energy calculated structures are summarized in Table S2. Cartesian coordinates of the lowest energy structures are shown in Table S3.

1.3. Breast Cancer Zebrafish Xenograft Experiments

Animal Care and Handling - *In vivo* experiments were performed in zebrafish (*Danio rerio*) lines *Tg(Fli1:eGFP)* and *Tg(mpx:eGFP)*, which were maintained and handled in accordance with European Animal Welfare Legislation and Champalimaud Fish Platform Program.

Maximum tolerated concentration assay - To assess the maximum tolerated concentration (MTC) of each compound in zebrafish larvae, a MTC assay was performed using the *in vitro* viability assays as reference for the tested concentrations. β-lapachone and Propargyl-Lap were diluted in the E3 medium and PdI_2-NPs were either injected into the PVS or also diluted in E3. Groups of 30-40 3dpf non-injected zebrafish larvae were exposed to different concentrations of the compounds during 3 consecutive days, with the E3/drug being renewed every day. Toxicity was analyzed daily by counting the total number of dead larvae and checking for the presence of morphologic changes such cardiac edemas and curved tails. Propargyl-Lap was tested alone and in combination with PdI_2-NPs. The tested concentrations of the compounds, in different combinations, were the following: β-lapachone: 0.1 µM, 0.5 µM, 1 µM, 2.5 µM, 5 µM; Propargyl-Lap: 5 µM, 10 µM, 50 µM, 100 µM; PdI_2-NPs: a) diluted in E3 - 15 µM, 25 µM, 50 µM, 100 µM; b) injected into the PVS - 5 µM, 12.5 µM. A DMSO control equivalent to the highest compound concentration was always tested.

Cell Culture – The human breast cancer cell line Hs578T:tdTomato (originally from American Type Culture Collection) was cultured in filtered high glucose Dulbecco's Modified Eagle Medium (DMEM) (Biowest) supplemented with 10% Fetal Bovine Serum (FBS) (Sigma-Aldrich) and 1% Penicillin-Streptomycin (P/S) 10,000 U/mL (Hyclone). Cells were maintained in an incubator (inCu Safe) with a humidified atmosphere containing 5% CO_2 at 37 °C.

Zebrafish Xenograft Injection - Zebrafish larvae were anesthetized with Tricaine 1X and Hs578T:tdTomato fluorescent cancer cells (~800 cells/xenograft) were microinjected either alone or together with the PdI_2-NPs (5 µM) into the perivitelline space (PVS) of 48 hours post fertilization (hpf)

anesthetized zebrafish larvae. After injection, xenografts were left on Embryonic (E3) medium (5 mM NaCl, 0.17 mM KCl, 0.33 mM $CaCl_2$, 0.33 mM MgSO4, 10–5 % methylene blue) and maintained at 34 °C until the next day. At 24 hpi, successfully injected zebrafish xenografts were sorted into classes according to tumor size and then randomly distributed into the different treatment groups, with no less than 30 larvae per group.

Zebrafish Xenograft Drug Administration - Hs578T Xenografts were randomly distributed into treatment groups: DMSO (control), Propargyl-Lap (5 μM), or β-Lapachone (1 μM). Propargyl-Lap and β-Lapachone were resuspended in DMSO and then diluted in E3 medium. Daily, the media was renewed and dead xenografts were removed.

Immunofluorescence - At the end of the assay, zebrafish xenografts were sacrificed with a Tricaine 25X overdose and fixed in 4% formaldehyde (PFA) overnight, followed by storage in methanol at −20 °C. Whole-mount immunofluorescence was performed starting with hydration through a methanol series (75% > 50% > 25%). Next, xenografts were permeabilized with 0.1% (w/v) Triton in PBS and blocked with a mixture of PBS 1X, BSA, DMSO, Triton 1% (w/v), and goat serum, for 1 h at room temperature. The xenografts were then incubated with primary antibody - anti-cleaved caspase-3 (rabbit, Cell Signaling, 1:100) – overnight, followed by another overnight incubation with secondary antibody - anti-rabbit IgG 650 (goat, Dylight, 1:400) - and 50 μg/ml DAPI (for nuclear counterstaining). Washing was performed between all steps. Stained xenografts were then mounted between two coverslips using Mowiol mounting media (Sigma), allowing for double side imaging.

Imaging and Quantification - All images were obtained using a Zeiss LSM 710 fluorescence confocal microscope, with a 5 µm interval using the z-stack function. Generated images were processed using the FIJI/ImageJ software. Some of the acquired z-stacks were projected using maximum intensity projection. Number of cells was quantified with the cell counter plugin. Tumor size and activated caspase-3 was quantified according to Póvoa et al.[8]

Statistical Analysis - Statistical analysis was performed using the GraphPad Prism software version 8.0.1. All data was challenged by two normality tests – the D'Agostino & Pearson and the Shapiro-Wilk normality tests. Since all datasets passed the normality tests, data was analyzed by an ordinary one-way ANOVA with multiple comparisons. For all the statistical analysis, P-value (P) corresponds to a two-tailed test with a confidence interval of 95%. Statistical differences were considered significant whenever P<0.05 and statistical output was represented by stars as follows: non-significant (ns)>0.05, *≤0.05, **≤0.01, ***≤0.001 and ****≤0.0001. All the graphs presented the results as average ± standard error of the mean (SEM). The ROUT method was used with a Q=1% to identify outliers, which were excluded from further analysis.

2. Synthetic Procedures and Compounds Characterization

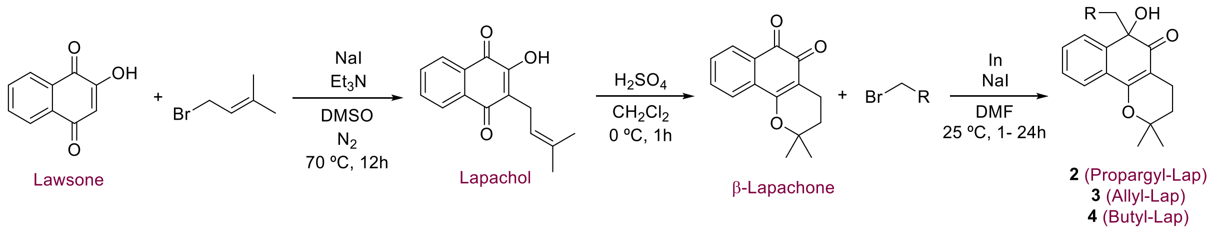

Lapachol

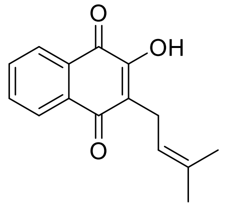

Lapachol was prepared using a modified version of a procedure reported in the literature.[9] Lawsone (2.8 g, 16.0 mmol), sodium iodide (2.4 g, 16 mmol) and triethylamine (2.8 mL, 20.0 mmol) was added into a 100 mL round bottom flask containing a magnetic stir bar in DMSO (40 mL). Then, 3,3-dimethylallyl bromide (2.5 mL, 20.0 mmol) was added to the solution and system was closed with a rubber septum and the mixture was stirred for 6 h at 70 °C. The reaction was diluted with water (50 mL) and extracted with CH_2Cl_2 (3x 50 mL). The organic phase was washed with a solution of sodium bicarbonate 10% in water (50 ml). The organic layer was dried over anhydrous Na_2SO_4, filtered and the solvent evaporated under reduced pressure and purified by silica gel column chromatography using 10% ethyl acetate in hexane solution as eluent, obtaining a yellow solid (1.68 g, 43% yield). ^{1}H NMR (200 MHz, $CDCl_3$, 25 °C) δ = 8.14 – 8.04 (m, 2H), 7.78 – 7.63 (m, 2H), 7.36 (s, 1H), 5.21 (t, J = 7.5 Hz, 1H), 3.31 (d, J = 7.4 Hz, 2H), 1.79 (s, 3H), 1.69 (s, 3H). ^{13}C NMR (50 MHz, $CDCl_3$, 25 °C) δ = 184.5, 181.7, 152.7, 134.8, 133.8, 132.9, 132.8, 129.4, 126.7, 126.0, 123.5, 119.6, 25.7, 22.6, 17.8. Agrees with data previously reported in the literature.[10]

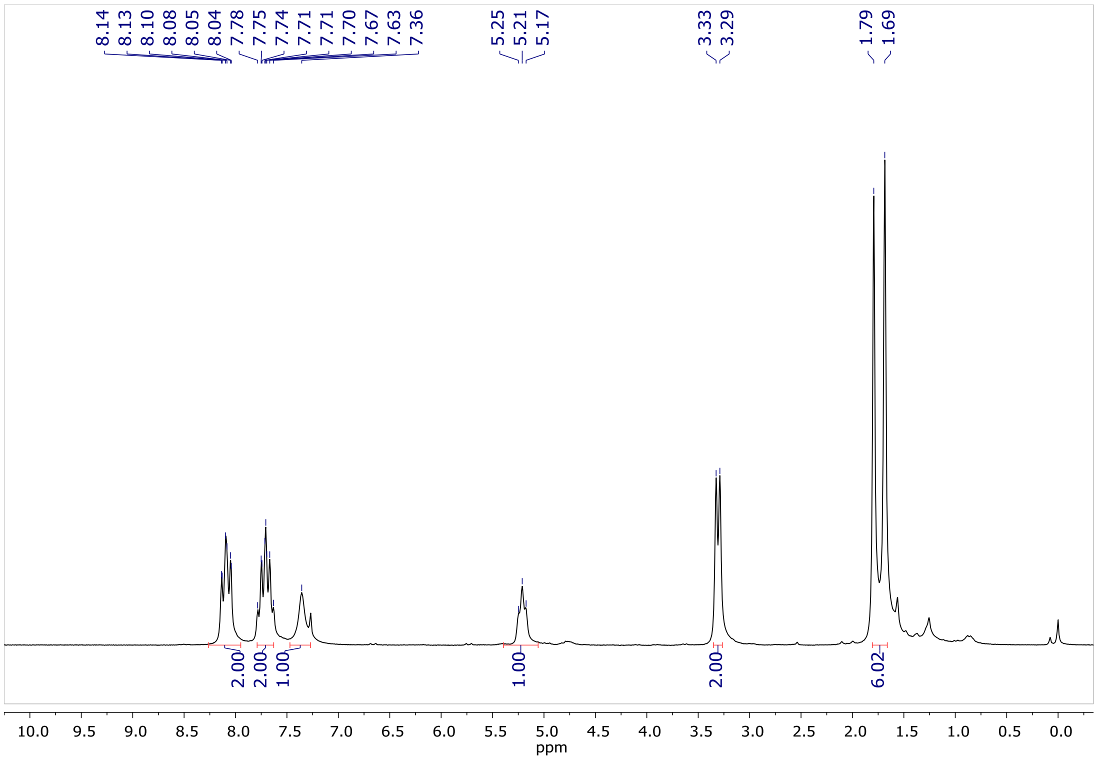

Figure S1. ^{1}H NMR spectrum of Lapachol (200 MHz, $CDCl_3$, 25 °C).

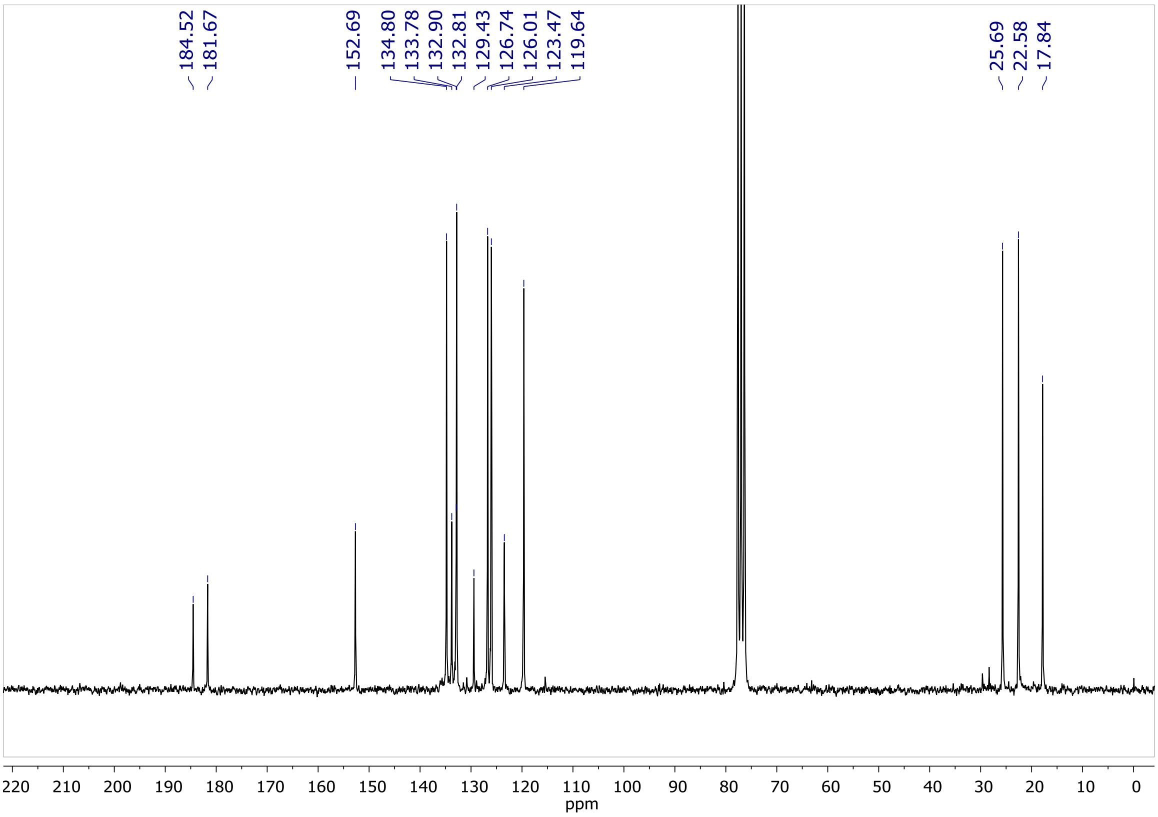

Figure S2. ^{13}C NMR spectrum of Lapachol (50 MHz, $CDCl_3$, 25 °C).

β-Lapachone (β-Lap, 1)

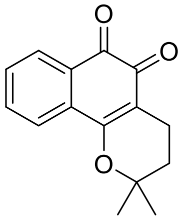

β-Lapachone was prepared using a modified version of a procedures reported in the literature.[10,11] A 100 mL round bottom flask, equipped with a stirrer bar, was charged with the Lapachol (1.5 g, 6.0 mmol) and dissolved in a mixture of CH_2Cl_2/H_2SO_4 (1:4, 40 mL) at 0 °C and stirred for 1 h at room temperature. The red solution was carefully poured into iced water and extracted with CH_2Cl_2 (3x 50 mL). The organic phase was washed with a solution of sodium bicarbonate 10% in water (2x 50 ml). The organic layer was dried over anhydrous Na_2SO_4, filtered and the solvent evaporated under reduced pressure and purified by silica gel column chromatography using 5-20% ethyl acetate in hexane solution as eluent, obtaining an orange solid (0.64 g, 43% yield). Melting point: 158-162 °C, Lit.[10] 153-155 °C. ^{1}H NMR (200 MHz, $CDCl_3$, 25 °C) δ = 8.06 (dd, *J* = 7.6, 1.2 Hz, 1H), 7.82 (dd, *J* = 7.7, 1.4 Hz, 1H), 7.65 (td, *J* = 7.6, 1.5 Hz, 1H), 7.50 (td, *J* = 7.5, 1.4 Hz, 1H), 2.58 (t, *J* = 6.6 Hz, 2H), 1.86 (t, *J* = 6.7 Hz, 2H), 1.47 (s, 6H). ^{13}C NMR (50 MHz, $CDCl_3$, 25 °C) δ = 179.8, 178.5, 162.0, 134.7, 132.6, 130.6, 130.1, 128.5, 124.0, 112.7, 79.2, 31.6, 26.7, 16.1. HRMS (ESI-TOF): $C_{15}H_{15}O_3$ (M + H) requires 243.1021/found: 243.1051. Agrees with data previously reported in the literature.[10]

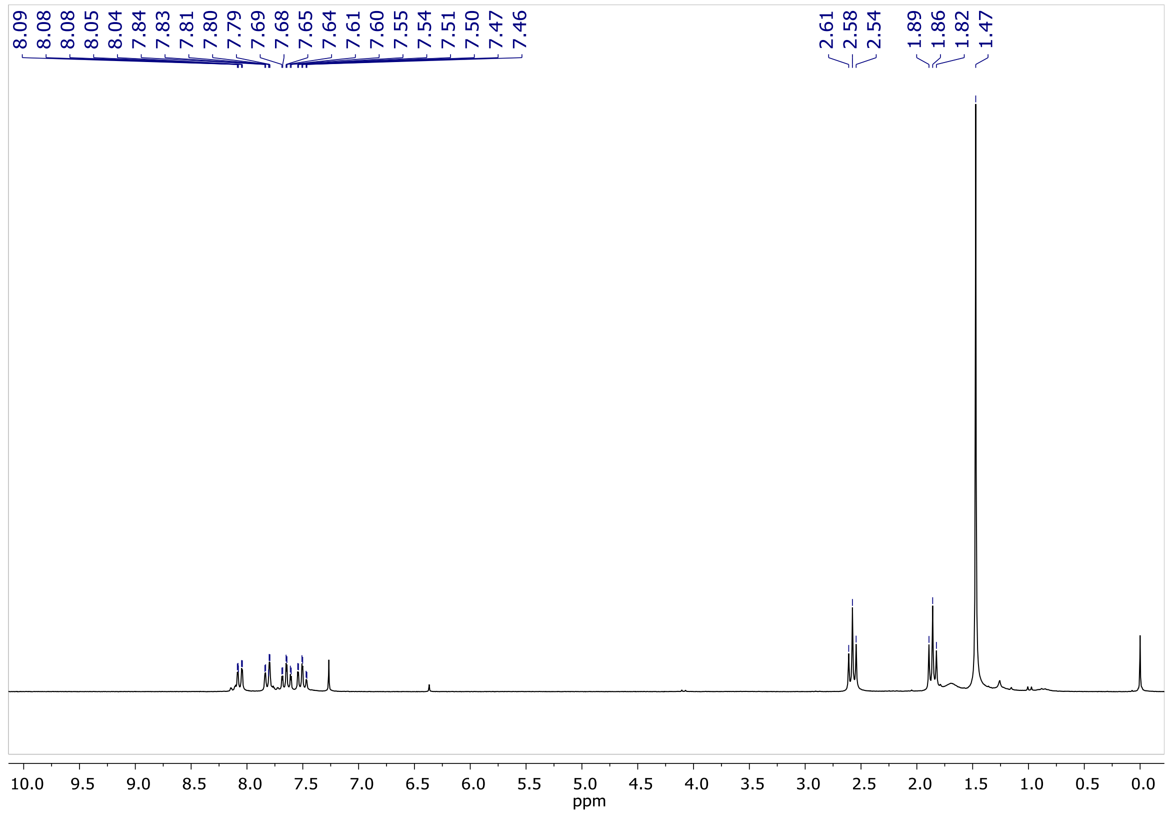

Figure S3. ^{1}H NMR spectrum of β-Lapachone (200 MHz, $CDCl_3$, 25 °C).

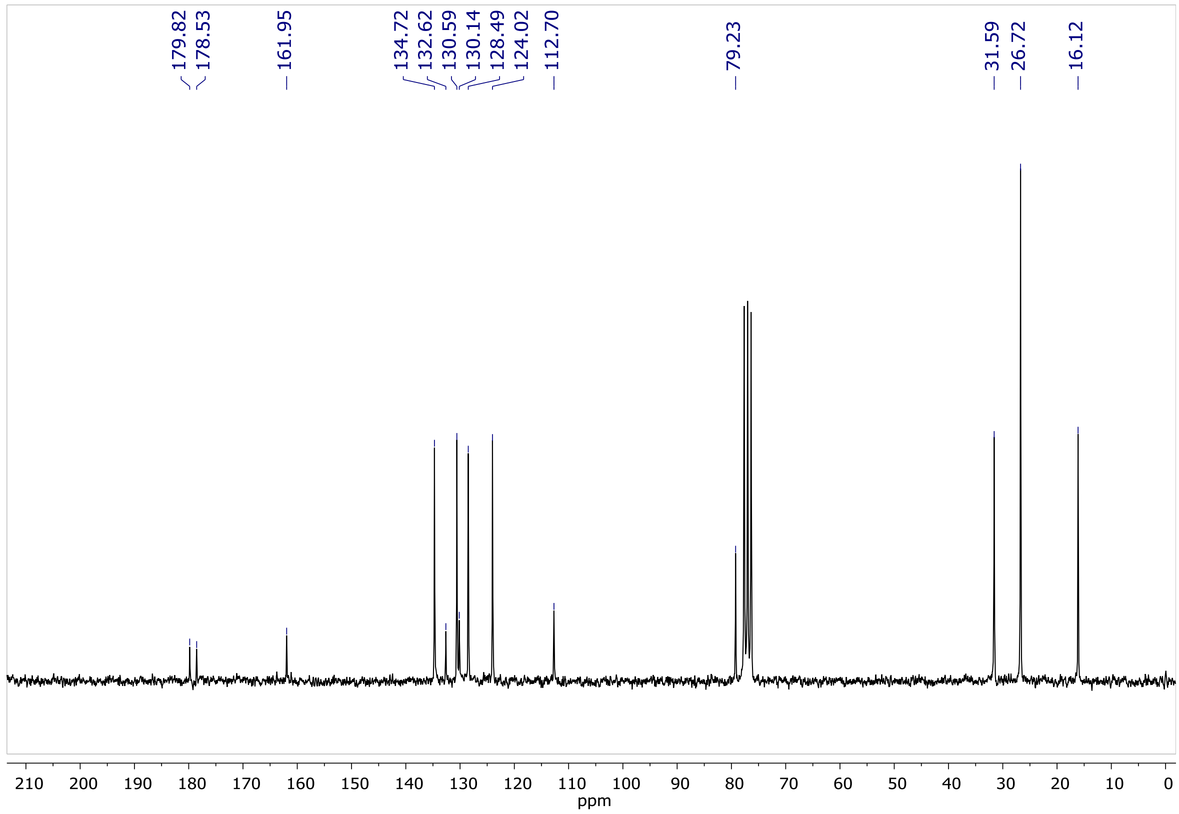

Figure S4. ^{13}C NMR spectrum of β-Lapachone (50 MHz, $CDCl_3$, 25 °C).

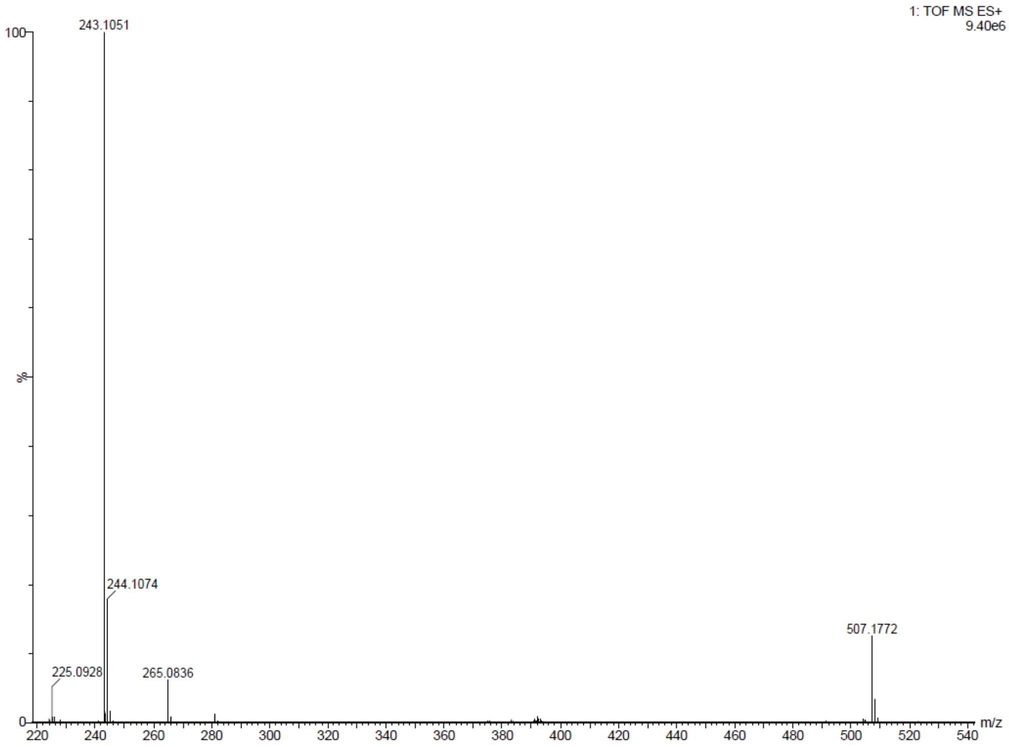

Figure S5. HRMS Spectrum of β-Lapachone.

Propargyl-Lap (2)

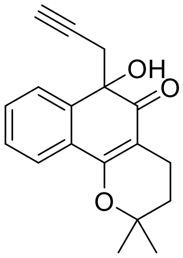

Propargyl-Lap was prepared using a modified version of a procedures reported in the literature.[12] β-Lapachone (0.242 g, 1.0 mmol), sodium iodide (0.232 g, 1.5 mmol) and indium powder (0.120 g, 1.05 mmol) was added into a 5 mL round bottom flask containing a magnetic stir bar in DMF (3 mL). Then, propargyl bromide (80% in toluene, 170 μL, 1.5 mmol) was added to the solution and system was closed with a rubber septum. The reaction was ultrasonicated for 1 min and stirred for 30 min at 25 °C and quenched with a few drops of HCl (5.0 M). The reaction was diluted with water (40 mL) and extracted with ethyl acetate (3x 25 mL). The organic phase was washed with a solution of brine (25 mL) and water (25ml). The organic layer was dried over anhydrous Na_2SO_4, filtered and the solvent evaporated under reduced pressure and purified by silica gel column chromatography using 5-10% ethyl acetate in hexane solution as eluent, obtaining a colorless solid (0.141 g, 50% yield). Melting point: 119-120 °C. ^{1}H NMR (700 MHz, $CDCl_3$, 25 °C) δ = 7.72 (d, *J* = 7.8 Hz, 1H), 7.64 (d, *J* = 7.7 Hz, 1H), 7.39 (td, *J* = 7.6, 1.2 Hz, 1H), 7.30 (td, *J* = 7.7, 1.2 Hz, 1H), 4.06 (s, 1H), 2.61 – 2.57 (m, 3H), 2.34 – 2.29 (m, 1H), 1.91 (t, *J* = 2.7 Hz, 1H), 1.80 (dt, *J* = 13.4, 5.7 Hz, 1H), 1.72 – 1.67 (m, 1H), 1.40 (s, 3H), 1.35 (s, 3H). ^{13}C NMR (176 MHz, $CDCl_3$, 25 °C) δ = 199.4, 162.7, 140.9, 130.3, 127.9, 127.4, 125.5, 123.4, 106.4, 78.6, 78.4, 75.8, 72.6, 37.2, 32.0, 27.8, 25.8, 16.0. HRMS (ESI-TOF): $C_{18}H_{19}O_3$ (M + H) requires 283.1334/found: 283.1330.

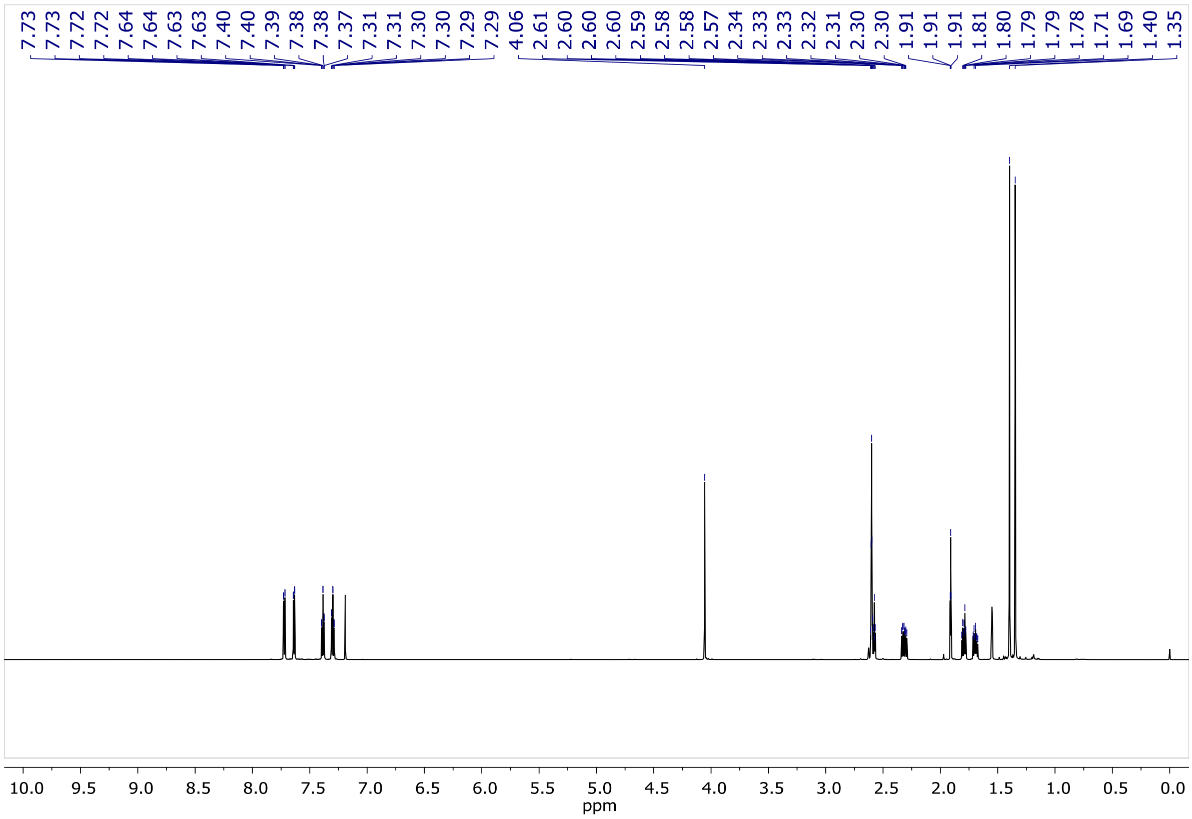

Figure S6. ^{1}H NMR of Propargyl-Lap (700 MHz, $CDCl_3$, 25 °C).

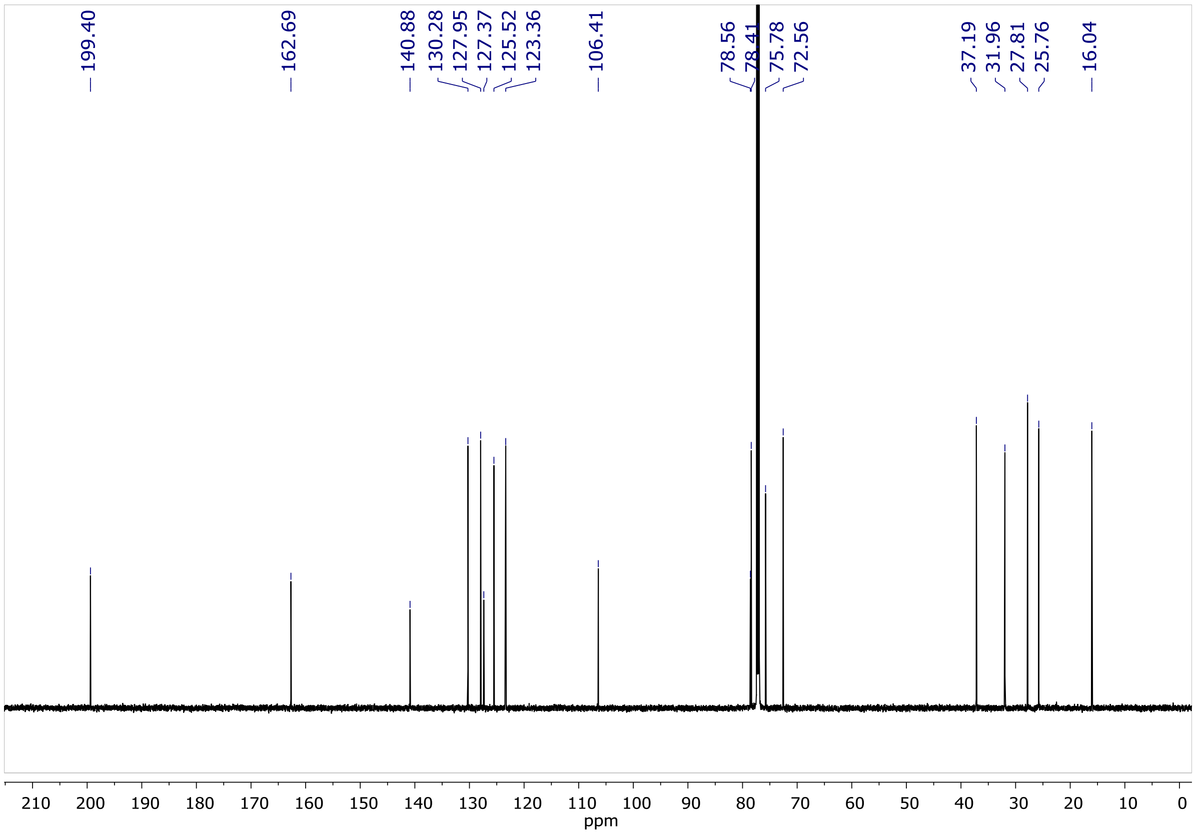

Figure S7. ^{13}C NMR of Propargyl-Lap (176 MHz, $CDCl_3$, 25 °C).

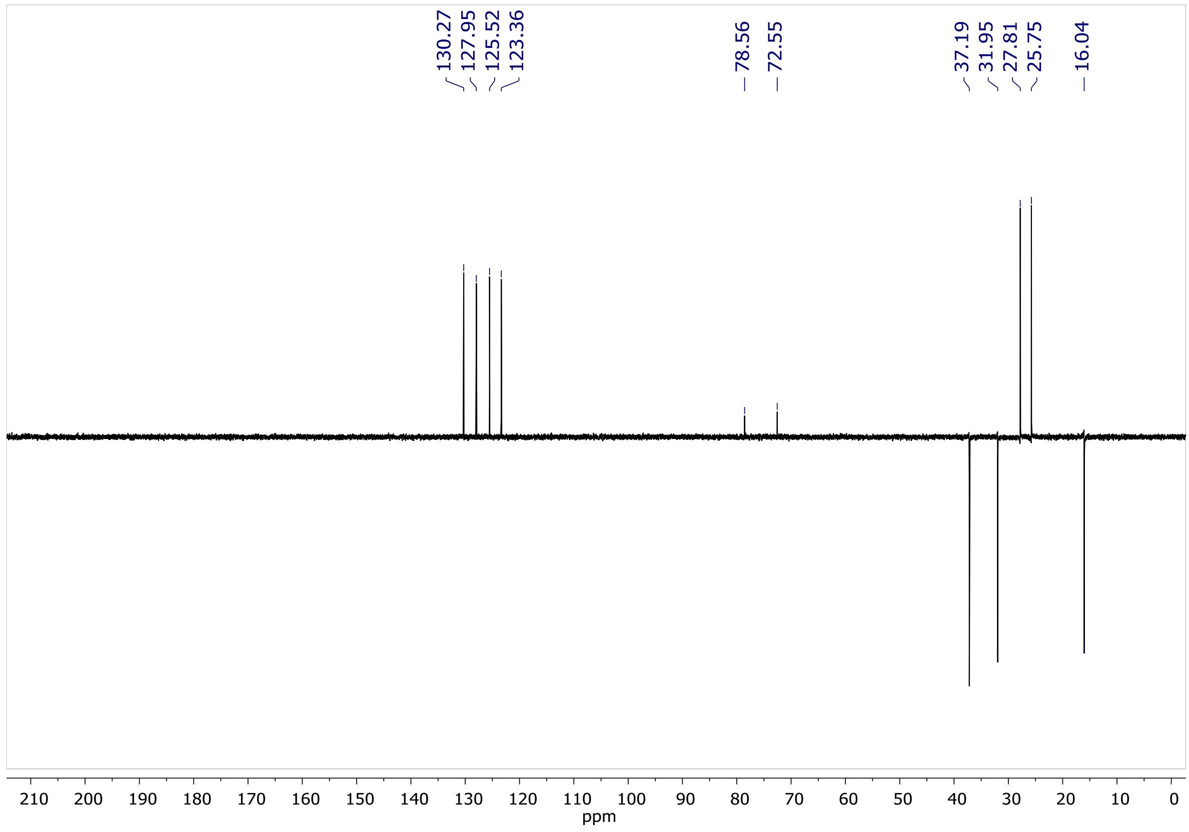

Figure S8. DEPT-135 NMR spectrum of Propargyl-Lap (176 MHz, $CDCl_3$, 25 °C).

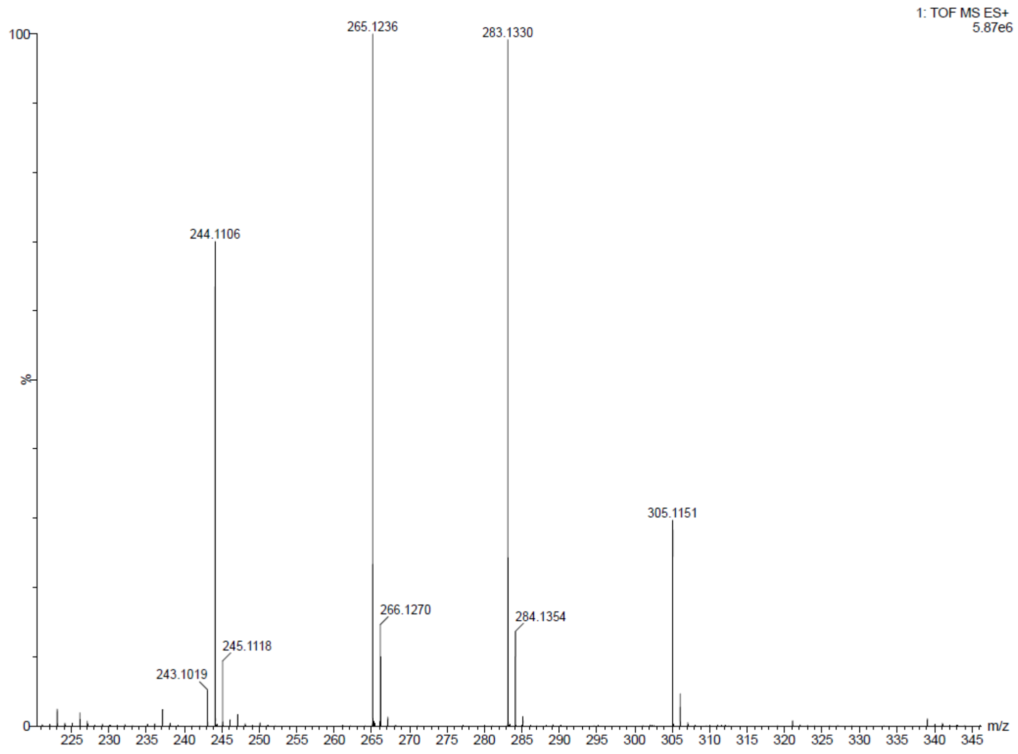

Figure S9. HRMS spectrum of Propargyl-Lap.

Allyl-Lap (3)

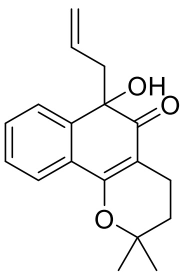

Allyl-Lap was prepared with the same procedure described for Propargyl-Lap, the reaction time was extended to 2 hours. White solid (0.232 g, 81% yield). Melting point: 75-77 °C. ^{1}H NMR (200 MHz, $CDCl_3$, 25 °C) δ = 7.77 (d, *J* = 7.8 Hz, 1H), 7.64 (d, *J* = 7.6 Hz, 1H), 7.44 (td, *J* = 7.6, 1.2 Hz, 1H), 7.33 (td, *J* = 7.5, 1.2 Hz, 1H), 5.61 – 5.40 (m, 1H), 5.03 – 4.92 (m, 1H), 4.92 – 4.76 (m, 1H), 3.93 (s, 1H), 2.63 (dt, *J* = 17.5, 6.0 Hz, 1H), 2.50 (d, *J* = 7.4 Hz, 2H), 2.39 – 2.23 (m, 1H), 1.96 – 1.68 (m, 2H), 1.45 (s, 3H), 1.41 (s, 3H). ^{13}C NMR (50 MHz, $CDCl_3$, 25 °C) δ = 201.1, 162.0, 142.1, 132.0, 130.1, 127.5, 127.0, 125.5, 123.3, 118.8, 106.5, 78.1, 77.5, 51.6, 32.0, 27.6, 25.9, 15.9. HRMS (ESI-TOF): $C_{18}H_{21}O_3$ (M + H) requires 285.1490/found: 285.1499.

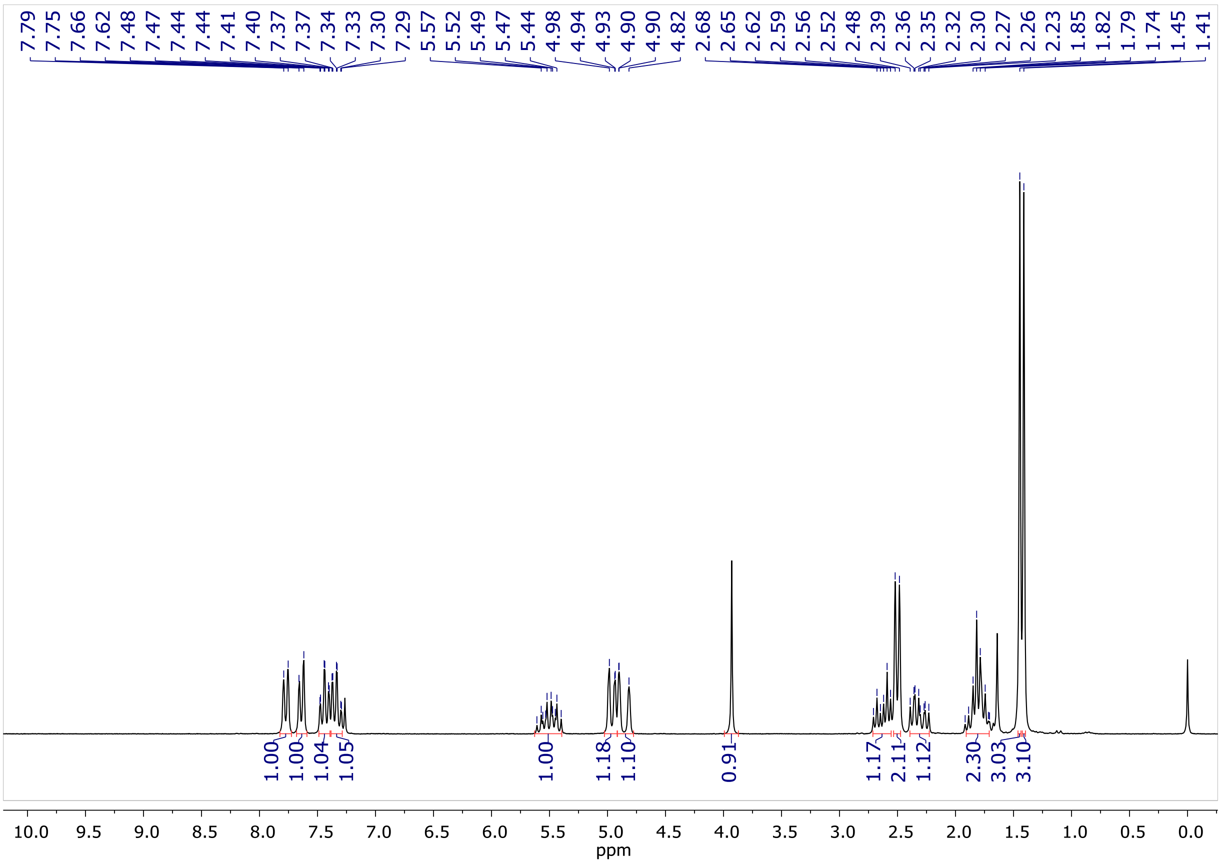

Figure S10. ^{1}H NMR spectrum of Allyl-Lap (200 MHz, $CDCl_3$, 25 °C).

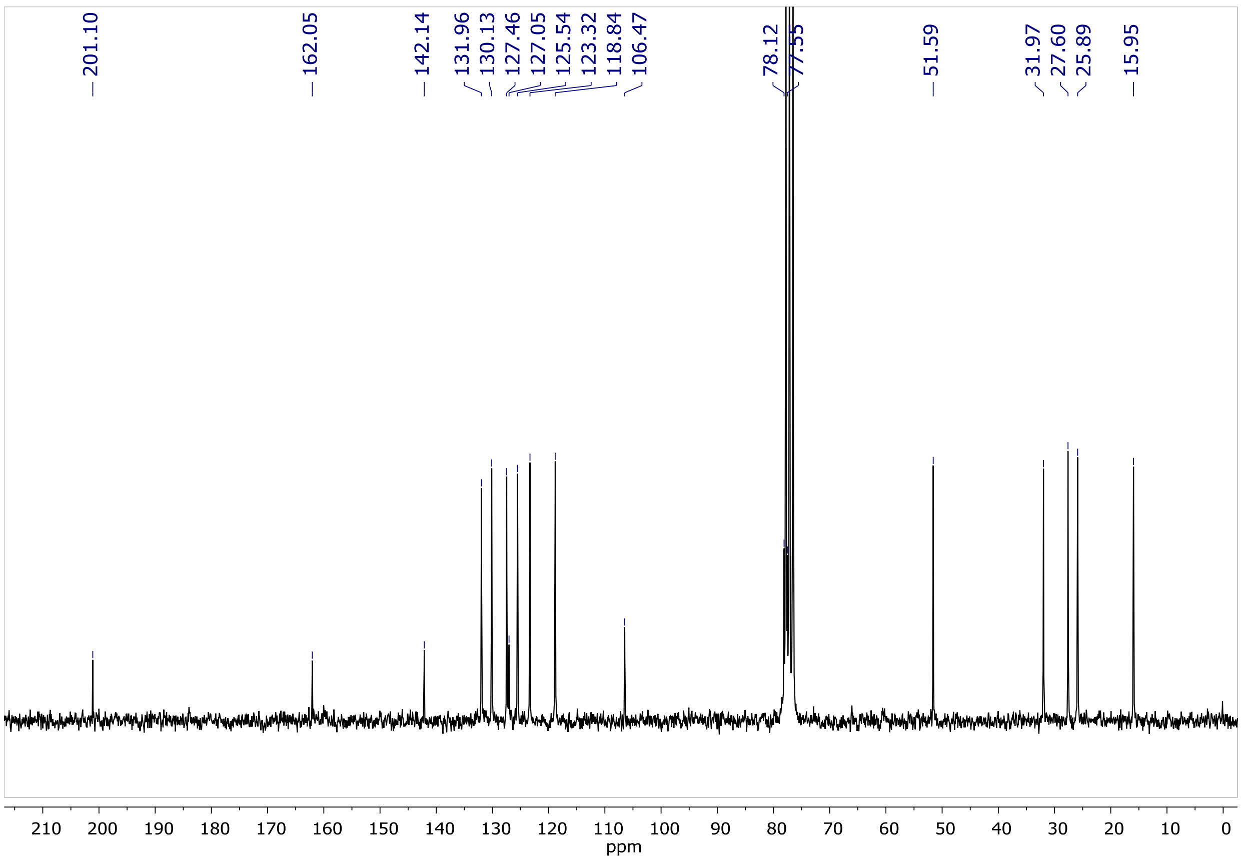

Figure S11. ^{13}C NMR spectrum of Allyl-Lap (50 MHz, $CDCl_3$, 25 °C).

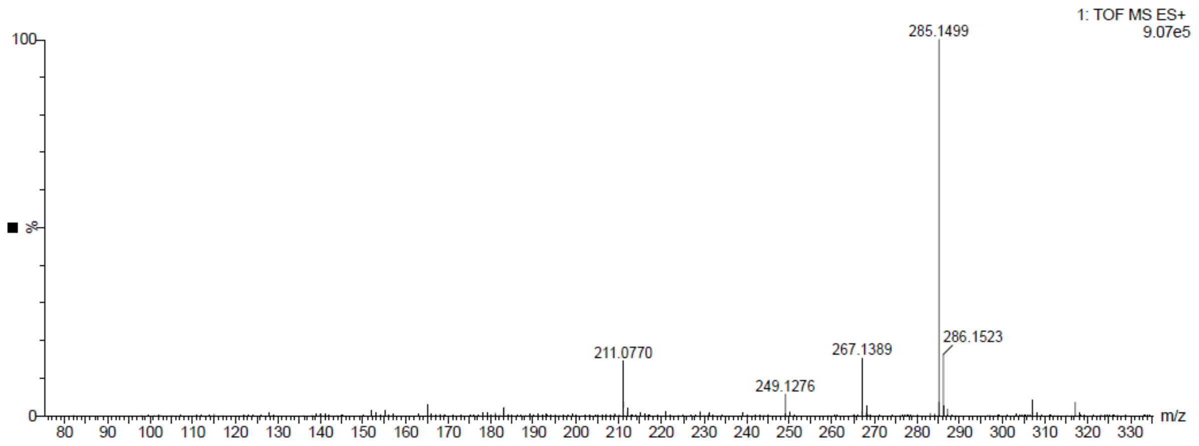

Figure S12. HRMS spectrum of Allyl-Lap.

Butyl-Lap (4)

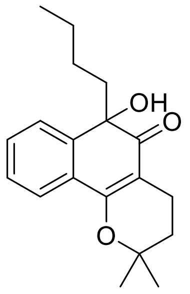

Butyl-Lap was prepared with the same procedure described for Propargyl-Lap, the reaction time was extended to 24 hours. Colorless solid (0.047 g, 16% yield). Melting point: 97-100 °C. ^{1}H NMR (200 MHz, CDCl$_3$, 25 °C) δ = 7.78 (d, *J* = 7.3 Hz, 1H), 7.63 (d, *J* = 7.5 Hz, 1H), 7.42 (td, *J* = 7.5, 1.3 Hz, 1H), 7.33 (td, *J* = 7.5, 1.3 Hz, 1H), 3.85 (s, 1H), 2.65 (dt, J = 17.4, 6.1 Hz, 1H), 2.35 (ddd, J = 17.5, 8.2, 6.2 Hz, 1H), 1.95 – 1.70 (m, 4H), 1.44 (s, 3H), 1.42 (s, 3H), 1.23 – 1.02 (m, 4H), 0.78 (t, *J* = 6.7 Hz, 3H). ^{13}C NMR (50 MHz, CDCl$_3$, 25 °C) δ = 202.2, 161.9, 143.1, 130.0, 127.3, 127.0, 125.5, 123.3, 106.3, 78.1, 47.0, 32.0, 27.5, 26.1, 26.0, 22.8, 16.1, 14.0. HRMS (ESI-TOF): $C_{19}H_{25}O_3$ (M + H) requires 301.1803/found: 301.1802.

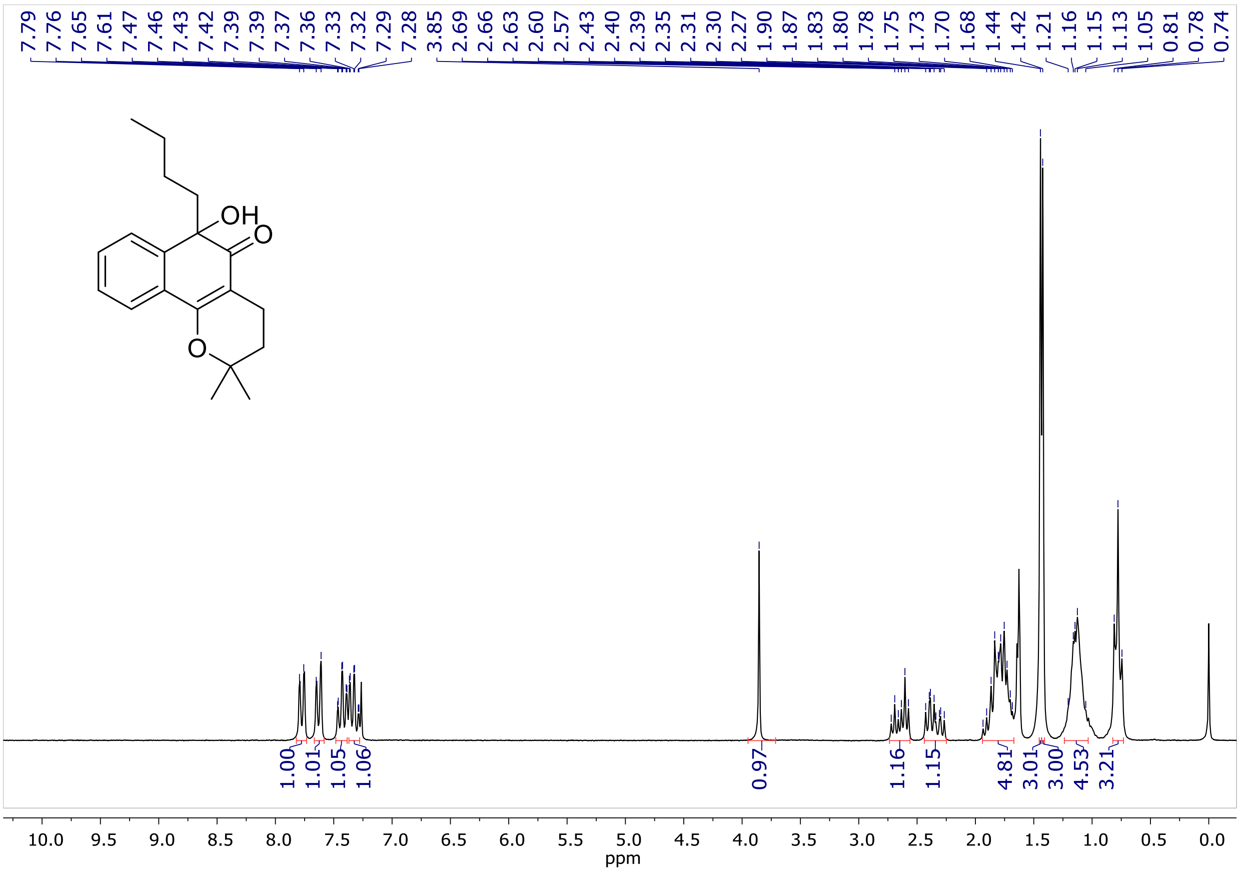

Figure S13. ^{1}H NMR spectrum of Butyl-Lap (200 MHz, CDCl$_3$, 25 °C).

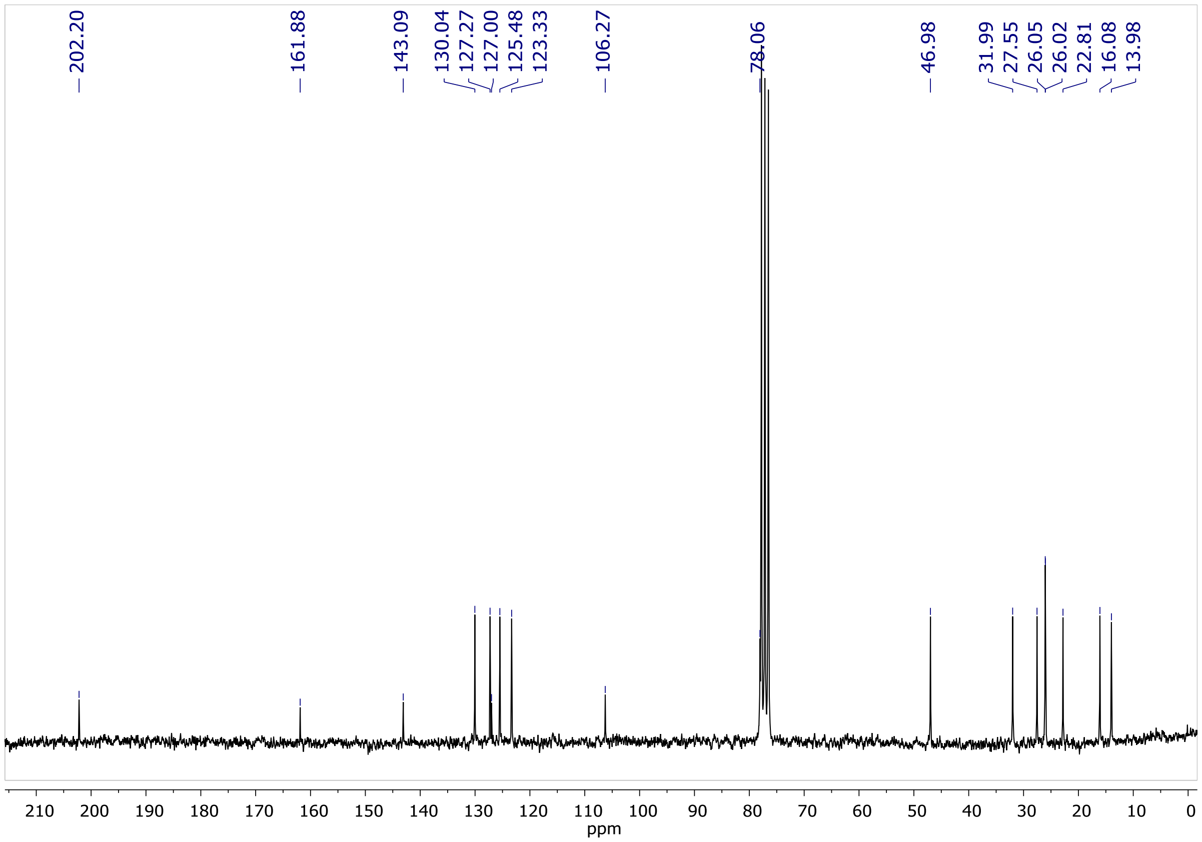

Figure S14. ^{13}C NMR spectrum of Butyl-Lap - (50 MHz, $CDCl_3$, 25 °C).

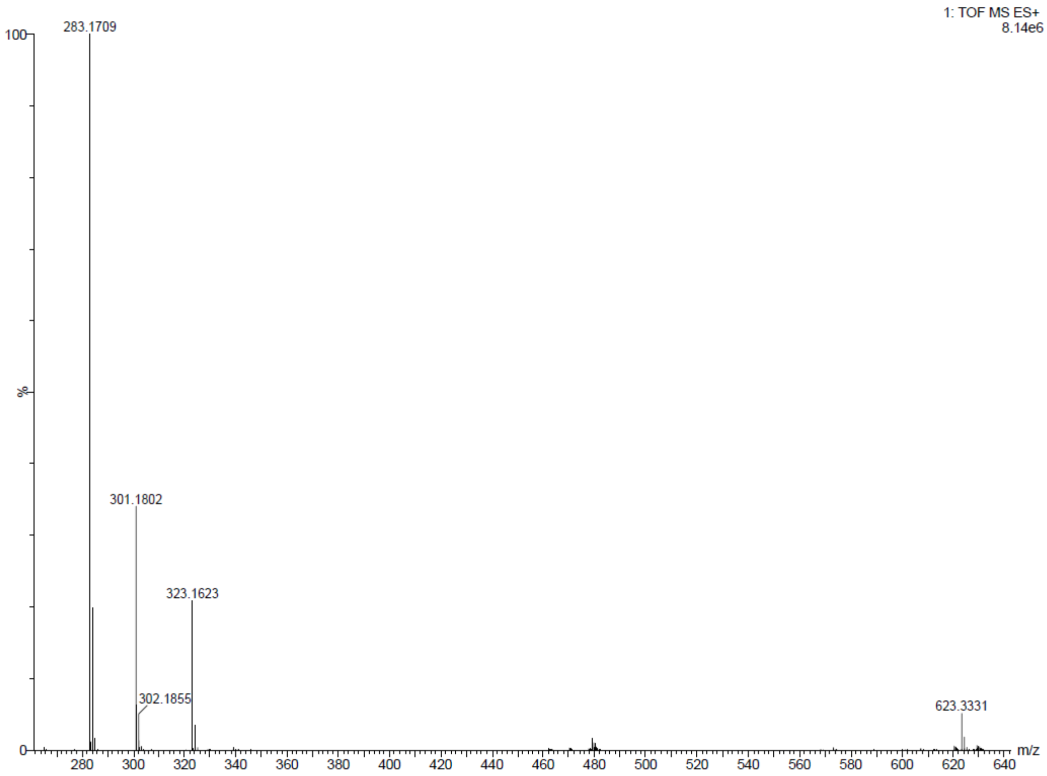

Figure S15. HRMS spectrum of Butyl-Lap.

PdI_2-NPs

The palladium (II) iodide nanoparticles were prepared according to the procedure described previously by us.[13] The synthesis consists in adding 3600 µL of a PVP solution (100 mM) and 1800 µL of $Pd(OAc)_2$ in acetonitrile solution (100 mM) to 12 mL of deionized water in the sample vials. After keeping the samples at room temperature for 10 minutes, 540 µL of a KI solution (500 mM) was added (1.5 eq. of KI). The suspension turned instantly dark brown. After 2 hours, 72 mL of acetone was added to the suspension. The sample was centrifuged for 20 minutes at 6000 RPM. The solid was dried in the oven at 60°C. The amount of palladium on the nanoparticles was quantified through Flame atomic absorption spectroscopy (FAAS). In average, the PdI_2-NPs contained 20% of palladium on the isolated solid.

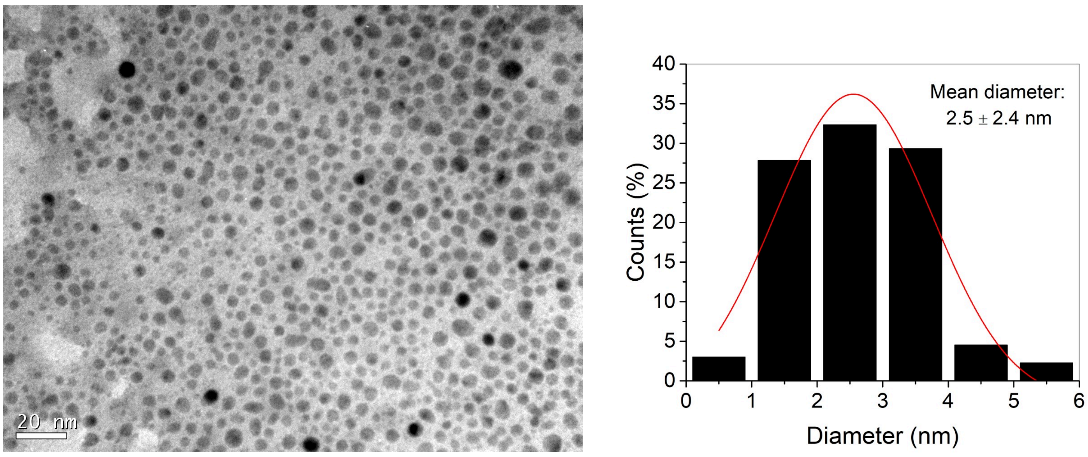

Figure S16. TEM micrograph and size distribution for the PdI_2-NPs

Pd(0)-NPs

The synthesis of the palladium (0) nanoparticles was conducted in similar manner for PdI_2-NPs, but KI solution was replaced by a solution of $NaBH_4$ (palladium reducing agent). The synthesis consists in adding 3600 µL of a PVP solution (100 mM) and 1800 µL of $Pd(OAc)_2$ in acetonitrile solution (100 mM) to 12 mL of deionized water in the sample vials. After keeping the samples at room temperature for 10 minutes, 540 µL of a $NaBH_4$ solution (500 mM) was added. The suspension turned instantly dark brown. After 2 hours, 72 mL of acetone was added to the suspension. The sample was centrifuged for 20 minutes at 6000 RPM. The solid was dried in the oven at 60°C. The amount of palladium on the nanoparticles was quantified through Flame atomic absorption spectroscopy (FAAS). In average, the PdI2-NPs contained 40% of palladium on the isolated solid.

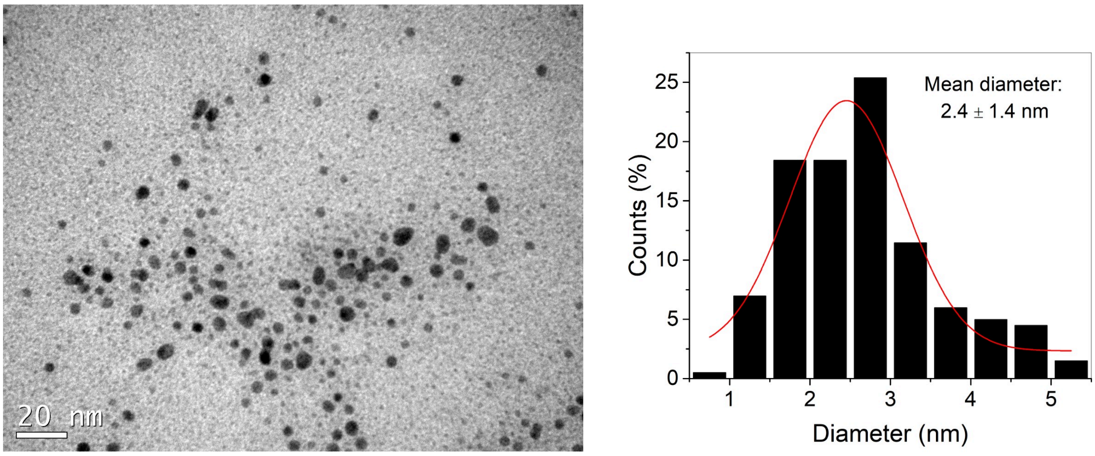

Figure S17. TEM micrograph and size distribution for the Pd(0)-NPs.

3. Reaction analysis of the palladium-mediated C-C decaging reaction of compounds 2-4 by LC-MS

The yield of C-C bond cleavage of the prodrugs by palladium under biologically relevant conditions was tested by incubating **2-4** (50 µM) at 37 °C in PBS (10% DMSO) with palladium sources. The reaction mixture was kept for 24 h at 37 °C under stirring and aliquots of the reaction (200 µL and diluted to 1000 µL with MeCN) were analyzed by LC-MS. Reactions were monitored by LC-MS with the observation of the protonated β-Lapachone ($[M+H]^+$, *m/z* 243, rt = 11.5 min). To determine yields, a calibration curve was constructed using product standards of known amounts of β-Lapachone (**Figure S18**).

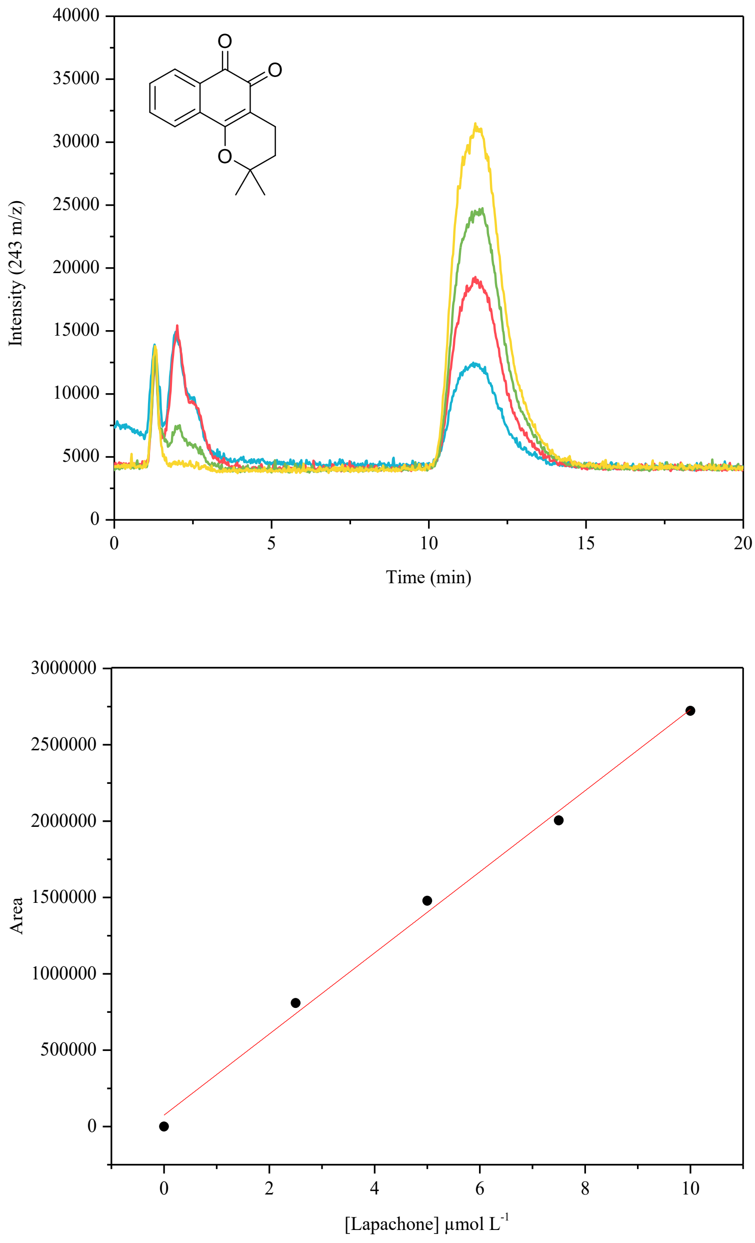

Figure S18. Calibration curve for β-Lapachone.

Table S1. Screening of palladium-mediated C-C decaging reaction of **2-4.**

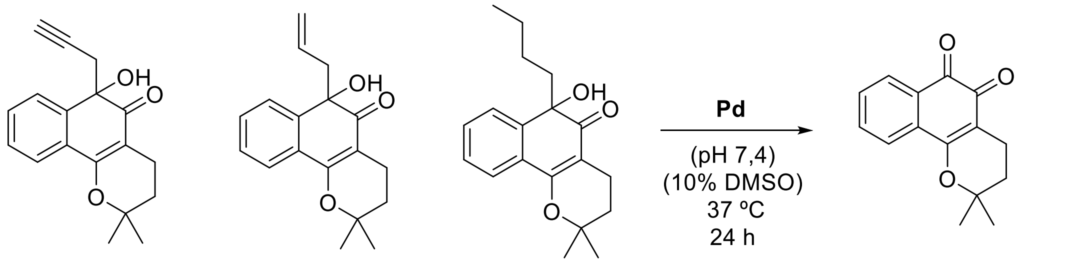

2 (Propargyl-Lap) **3** (Allyl-Lap) **4** (Butyl-Lap) **1** (β-Lapachone)

Entry	Pd	Protecting group	Eq. Pd	Medium[3]	Yield(%)
1	-	Propargyl	-	PBS	**0**
2	-	Allyl	-	PBS	**0**
3	Na_2PdCl_4	Butyl	5	PBS	**0**
4	Na_2PdCl_4	Propargyl	1	PBS	**21**
5	Na_2PdCl_4	Allyl	1	PBS	**13**
6	Na_2PdCl_4	Propargyl	0.5	PBS	**8**
7	Na_2PdCl_4	Allyl	0.5	PBS	**1**
8	Na_2PdCl_4	Propargyl	1	PB (I = 0 mM)	**23**
9	Na_2PdCl_4	Propargyl	1	H_2O	**56**
10	PdI_2-NPs	Propargyl	1	PBS	**66**
11	PdI_2-NPs	Allyl	1	PBS	**31**
12	PdI_2-NPs	Propargyl	1	PB (I = 0 mM)	**56**
13	Pd(0)-NPs	Propargyl	1	PBS	**4**
14	Pd(0)-NPs	Allyl	1	PBS	**0**
15	PdI_2	Propargyl	1	PBS	**0**
16	PdI_2 + PVP[1]	Propargyl	1	PBS	**0**
17	PdI_2-NPs + EDTA[2]	Propargyl	1	PBS	**8**
18	PdI_2-NPs	Propargyl	0.5	PBS	**32**

[1] 5 equiv of PVP.

[2] 10 equiv of EDTA.

[3] PBS = Phosphate buffer saline (140 mM of chloride), PB = Phosphate buffer (without chloride) with ionic strength (I) equal to zero.

4. LC-MS analysis for the C-C decaging of Propargyl-Lap mediated by PdI_2-NPs

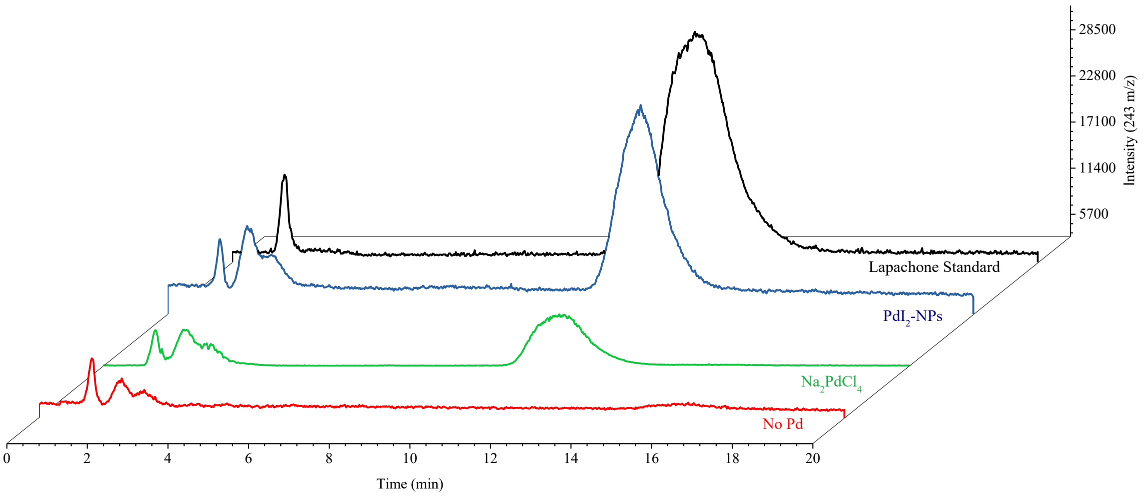

Figure S19. LC-MS chromatograms of C-C cleavage reaction of Propargyl-Lap mediated by PdI_2-NPs and Na_2PdCl_4. Reaction conditions: [Propargyl-Lap] = 50 µM, [Pd] = 50 µM, PBS (pH 7.4, 10 % DMSO), 24 h at 37°C.

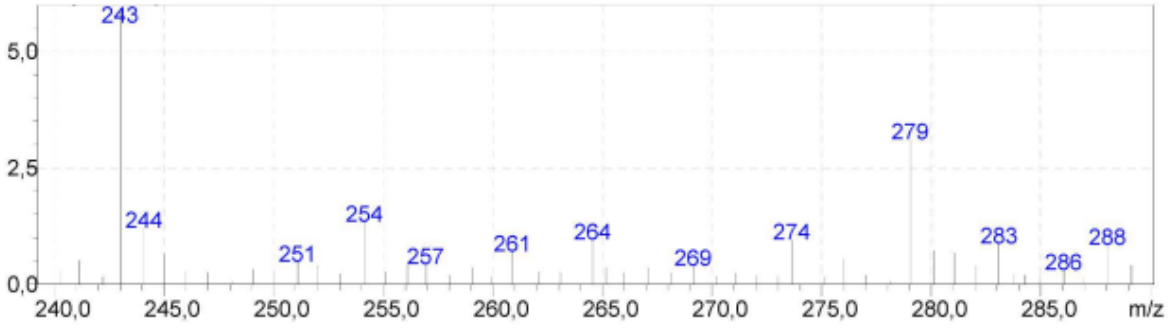

Figure S20. LC-MS spectrum of β-Lapachone ($[M+H]^+$, *m/z* 243, rt = 11.5 min).

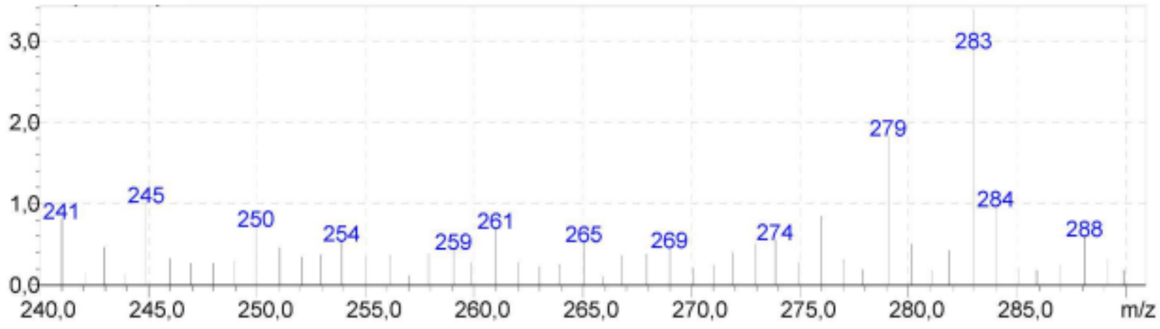

Figure S21. LC-MS spectrum of Propargyl-Lap ($[M+H]^+$, *m/z* 283, rt = 15.9 min).

5. ESI-MS(+) for the C-C decaging of Propargyl-Lap mediated by Na_2PdCl_4 in water

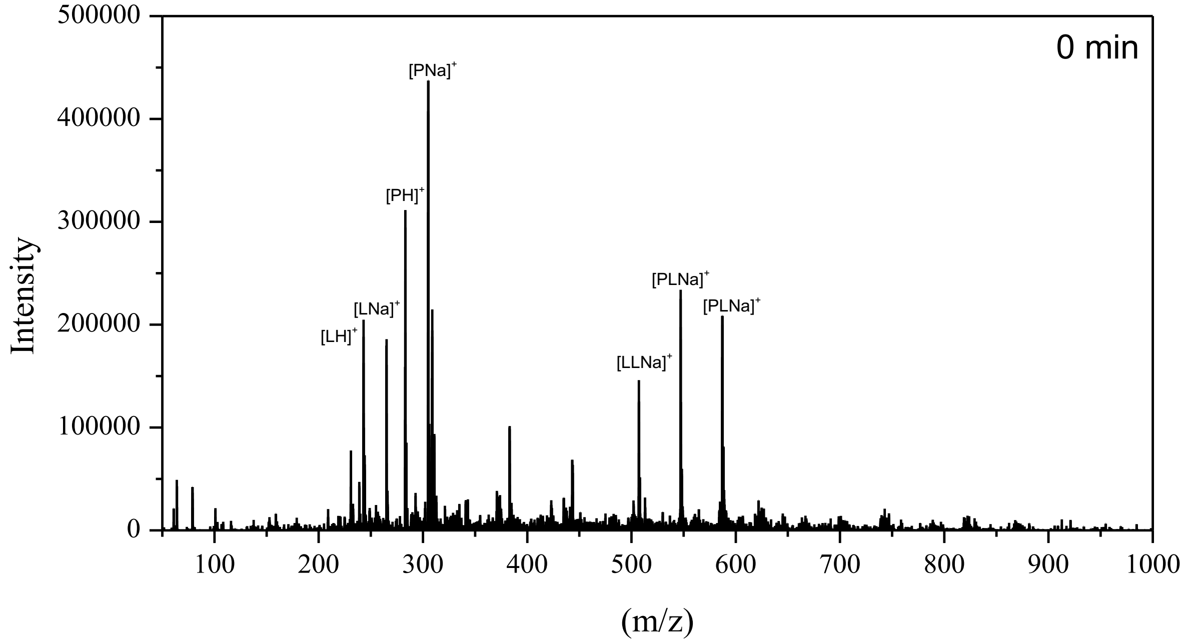

Figure S22. ESI-MS(+) of the reaction medium after the start of depropargylation of Propargyl-Lap mediated by Na_2PdCl_4 in water. P: Propargyl-Lap. L: β-Lapachone. Reaction conditions: [Propargyl-Lap] = 40 μM, [Na_2PdCl_4] = 20 μM at 37°C. (150 μL of reaction diluted in 150 μL of H_2O(MS)).

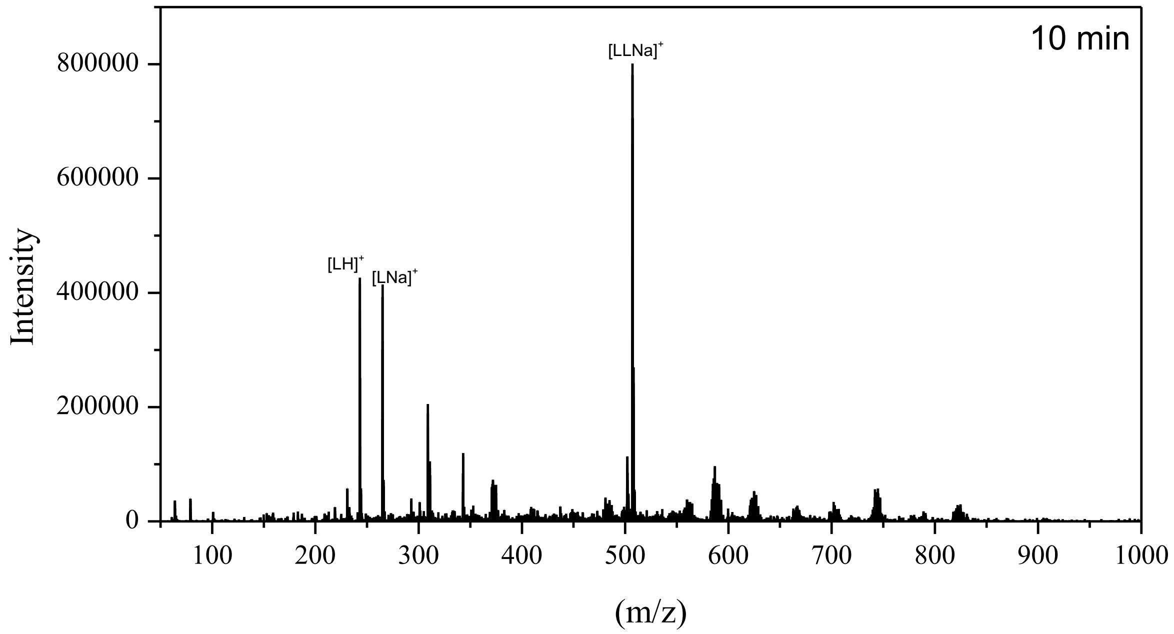

Figure S23. ESI-MS(+) of the reaction medium after 10 minutes of depropargylation of Propargyl-Lap mediated by Na_2PdCl_4 in water. P: Propargyl-Lap. L: β-Lapachone. Reaction conditions: [Propargyl-Lap] = 40 μM, [Na_2PdCl_4] = 20 μM at 37°C. (150 μL of reaction diluted in 150 μL of H_2O(MS)).

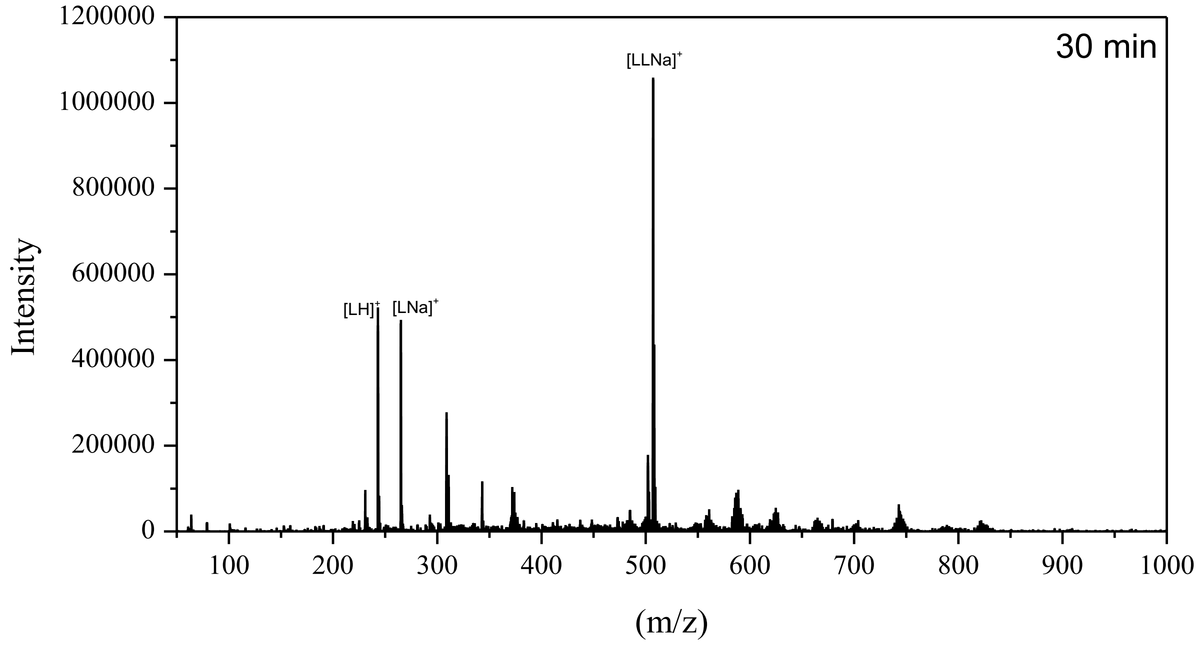

Figure S24. ESI-MS(+) of the reaction medium after 30 minutes of depropargylation of Propargyl-Lap mediated by Na_2PdCl_4 in water. P: Propargyl-Lap. L: β-Lapachone. Reaction conditions: [Propargyl-Lap] = 40 µM, [Na_2PdCl_4] = 20 µM at 37°C. (150 µL of reaction diluted in 150 µL of H_2O(MS)).

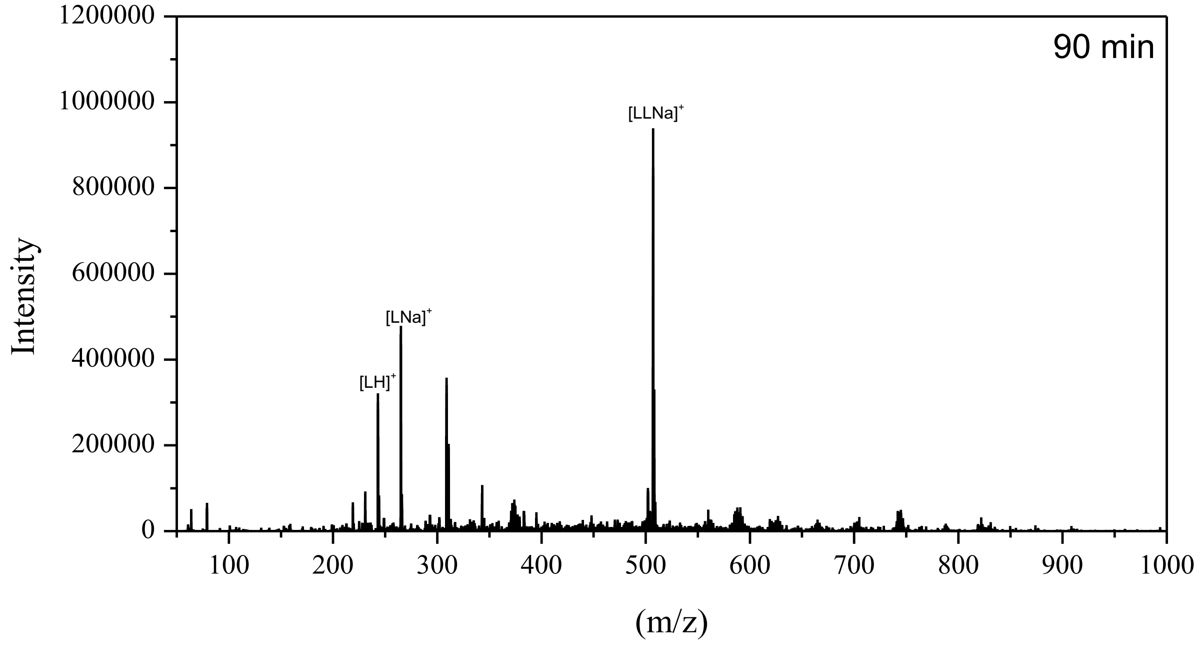

Figure S25. ESI-MS(+) of the reaction medium after 90 minutes of depropargylation of Propargyl-Lap mediated by Na_2PdCl_4 in water. P: Propargyl-Lap. L: β-Lapachone. Reaction conditions: [Propargyl-Lap] = 40 µM, [Na_2PdCl_4] = 20 µM at 37°C. (150 µL of reaction diluted in 150 µL of H_2O(MS)).

6. ESI-MS(+) for the C-C decaging of Allyl-Lap mediated by Na_2PdCl_4 in water

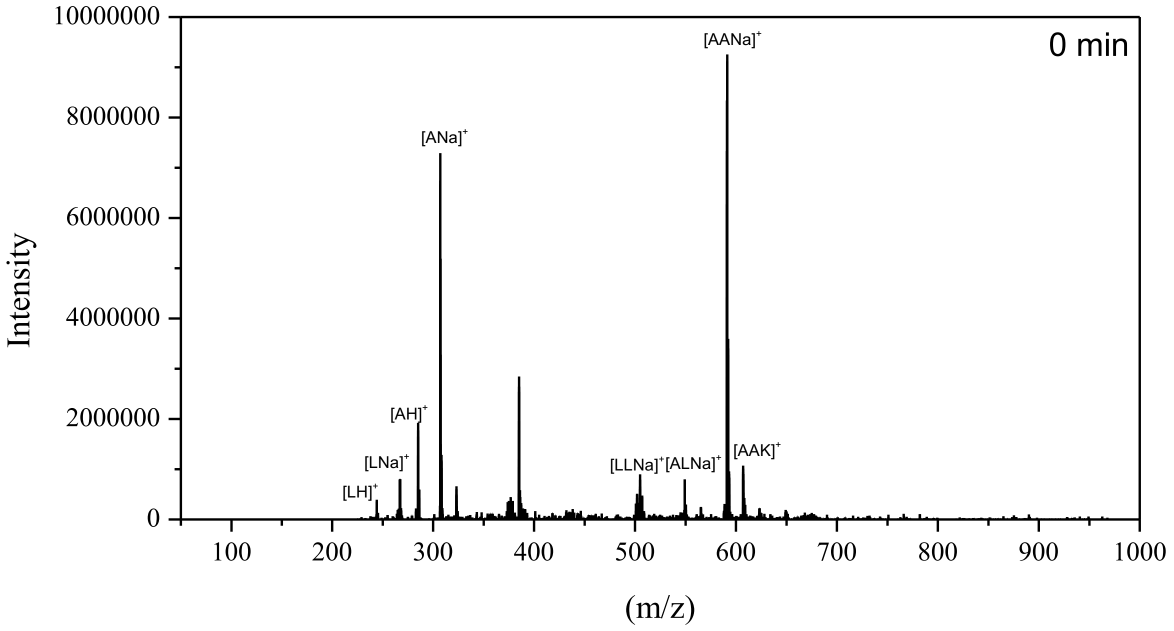

Figure S26. ESI-MS(+) of the reaction medium after the start of deallylation of Allyl-Lap mediated by Na_2PdCl_4 in water. A: Allyl-Lap. L: β-Lapachone. Reaction conditions: [Allyl-Lap] = 40 μM, [Na_2PdCl_4] = 20 μM at 37°C. (150 μL of reaction diluted in 150 μL of H_2O(MS)).

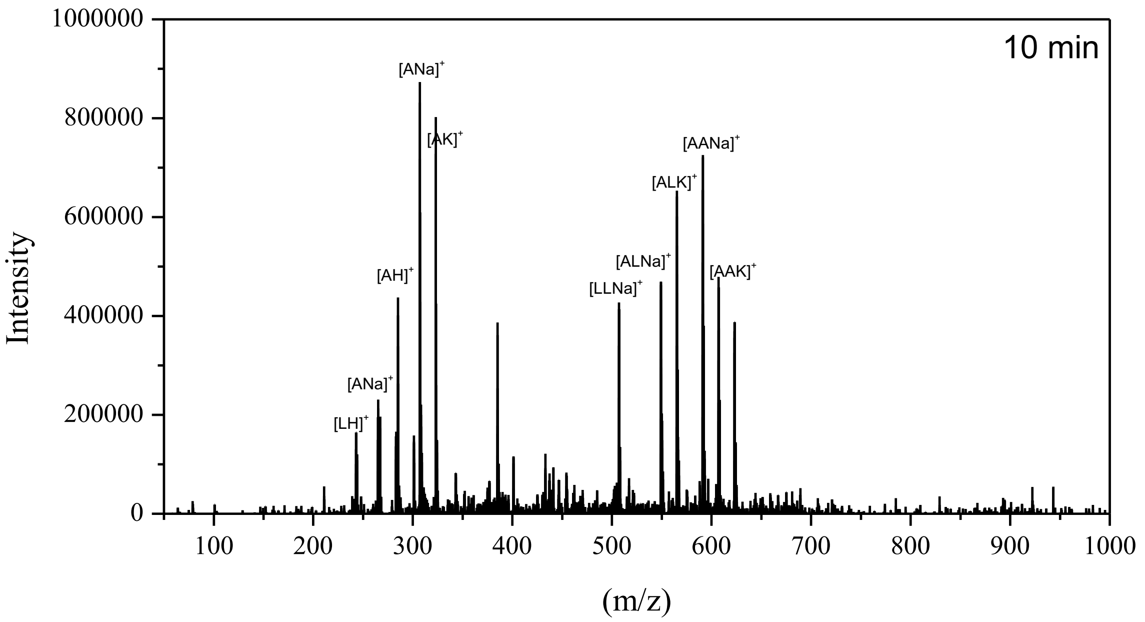

Figure S27. ESI-MS(+) of the reaction medium after 10 minutes of deallylation of Allyl-Lap mediated by Na_2PdCl_4 in water. A: Allyl-Lap. L: β-Lapachone. Reaction conditions: [Allyl-Lap] = 40 μM, [Na_2PdCl_4] = 20 μM at 37°C. (150 μL of reaction diluted in 150 μL of H_2O(MS)).

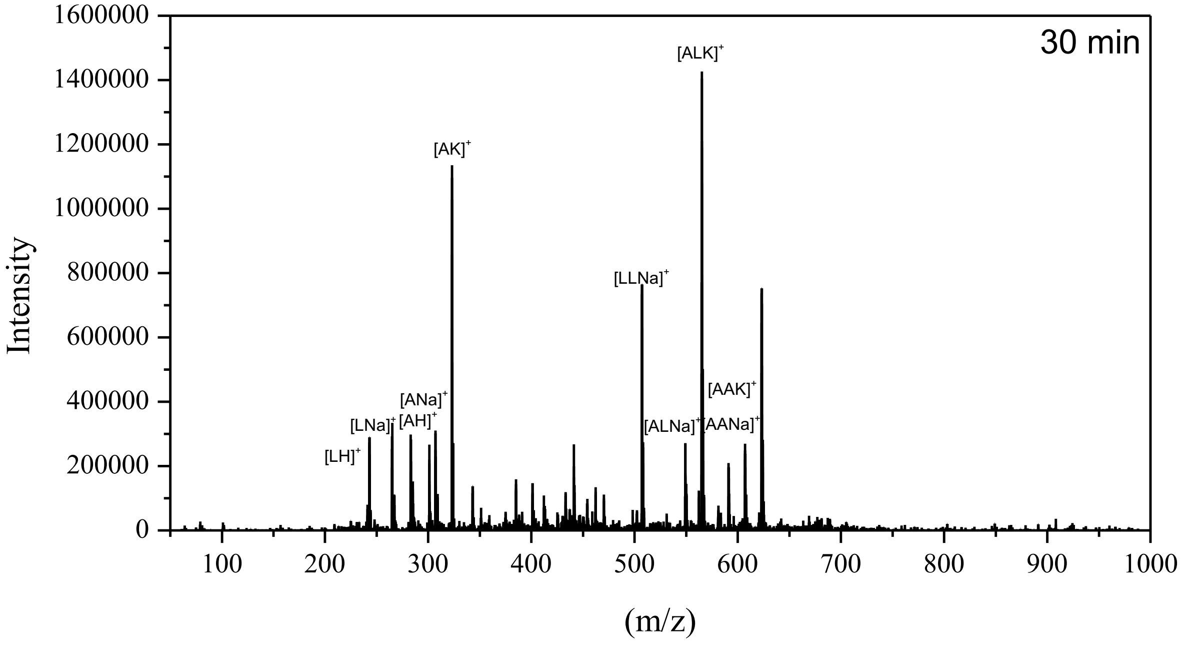

Figure S28. ESI-MS(+) of the reaction medium after 30 minutes of deallylation of Allyl-Lap mediated by Na_2PdCl_4 in water. A: Allyl-Lap. L: β-Lapachone. Reaction conditions: [Allyl-Lap] = 40 µM, [Na_2PdCl_4] = 20 µM at 37°C. (150 µL of reaction diluted in 150 µL of H_2O(MS)).

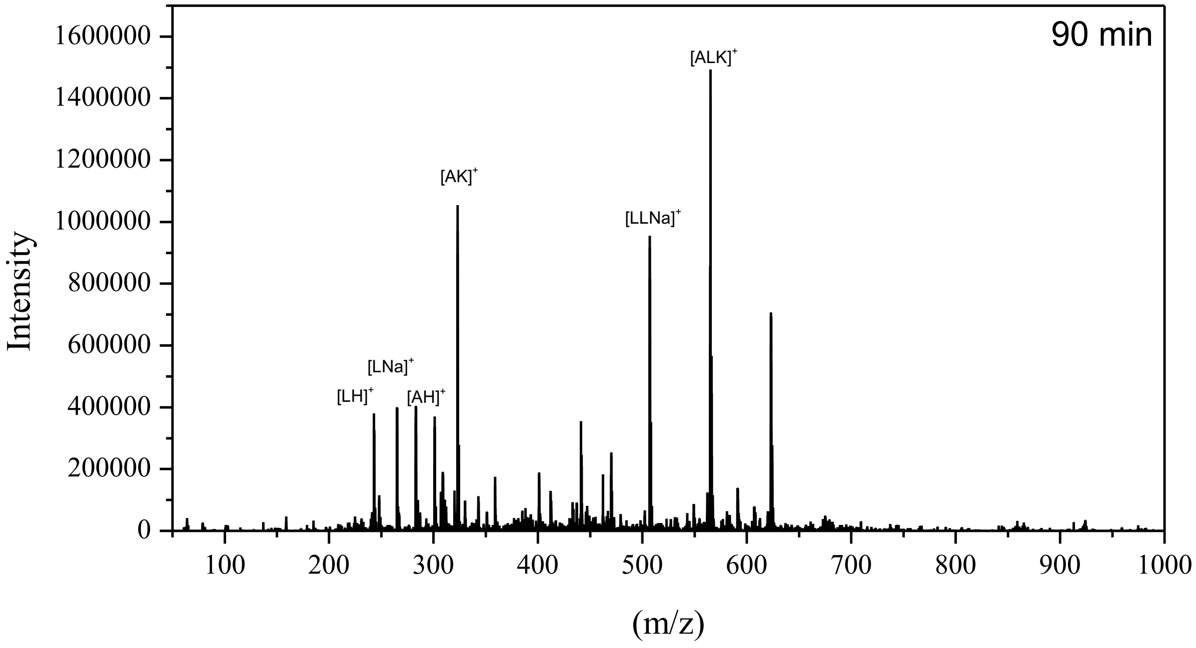

Figure S29. ESI-MS(+) of the reaction medium after 90 minutes of deallylation of Allyl-Lap mediated by Na_2PdCl_4 in water. A: Allyl-Lap. L: β-Lapachone. Reaction conditions: [Allyl-Lap] = 40 µM, [Na_2PdCl_4] = 20 µM at 37°C. (150 µL of reaction diluted in 150 µL of H_2O(MS)).

7. Quantum Mechanical data

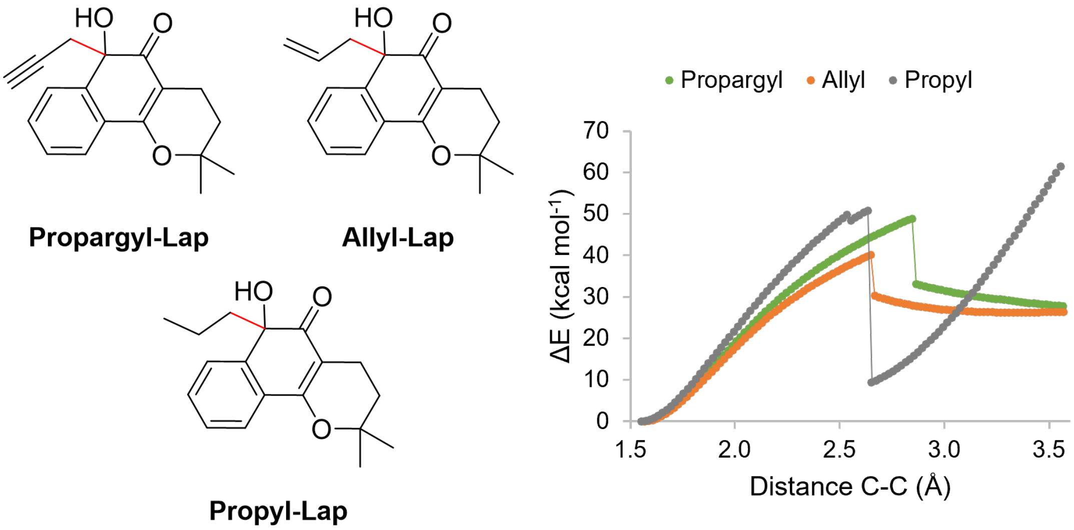

Figure S30. Potential energy surfaces (PES) calculated with PCM(H_2O)/M06-2X/6-31+G(d,p) for the elimination of neutral propargyl (green), allyl (orange), and propyl (grey) β-Lap derivatives. All the scans were started from the lowest energy conformer for each derivative. No elimination TS was found for any of the derivatives. Instead, the leaps on the PES correspond to an artificial shift of the propargyl, allyl, and/or propyl fragments to the adjacent carbonyl group.

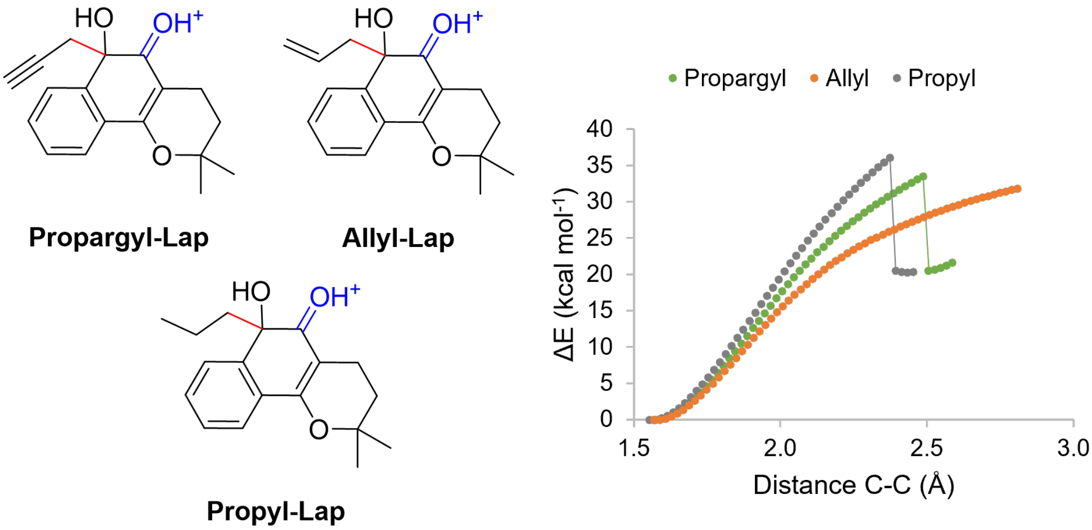

Figure S31. Potential energy surfaces (PES) calculated with PCM(H_2O)/M06-2X/6-31+G(d,p) for the elimination of the protonated carbonyl species for the propargyl (green), allyl (orange), and propyl (grey) β-Lap derivatives. All the scans were started from the lowest energy conformer for each derivative. No elimination TS was found for any of the derivatives. Instead, the leaps on the PES correspond to an artificial shift of the propargyl, allyl, and/or propyl fragments to the adjacent carbonyl group.

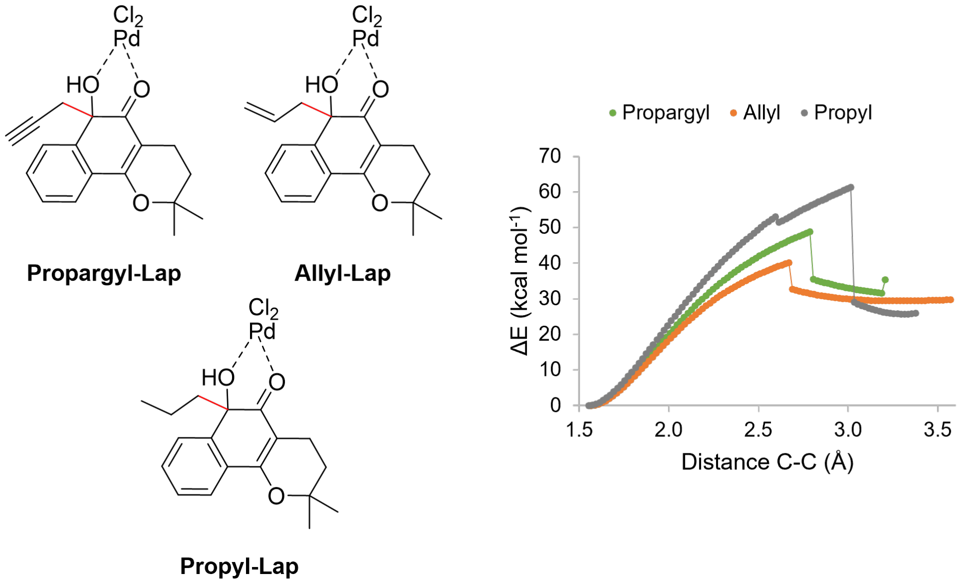

Figure S32. Potential energy surfaces (PES) calculated with PCM(H_2O)/M06-2X/6-31+G(d,p)+LanL2DZ(Pd) for the elimination of propargyl (green), allyl (orange), and propyl (grey) β-Lap derivatives with Pd(II) as a Lewis acid chelating the carbonyl and the hydroxyl groups. All the scans were started from the lowest energy conformer for each derivative. No elimination TS was found for any of the derivatives. Instead, the leaps on the PES correspond to an artificial shift of the propargyl, allyl, and/or propyl fragments to the adjacent carbonyl group.

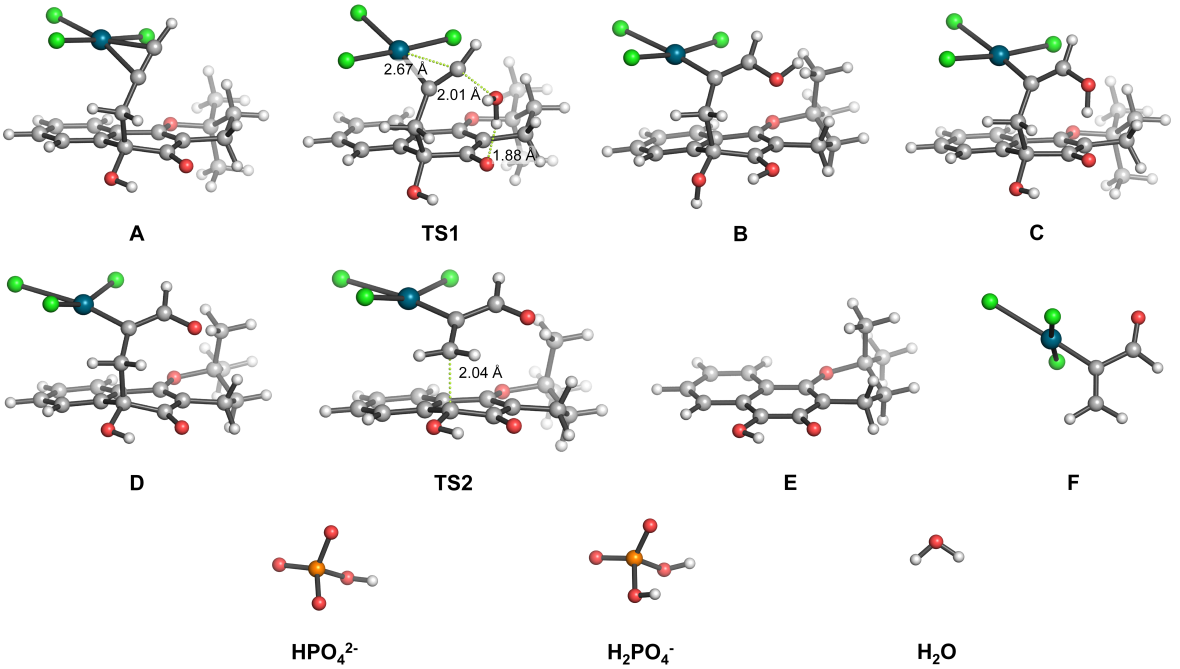

Figure S33. Geometries for the reactants (**A**), transition states (**TS1**,**2**), intermediates (**B**-**D**) and products (**E**,**F**) for the reaction pathway calculated with PCM(H_2O)/M06-2X/6-31+G(d,p)+LanL2DZ(Pd). Interatomic distances are given in angstroms. Breaking/forming bonds on the transition states are shown as dotted green lines.

Table S2. Energies, entropies, and lowest frequencies of the lowest energy structures calculated with PCM(H_2O)/ωB97x-D/6-311+G(2d,p)+LanL2DZ(Pd)//PCM(H_2O)/M06-2X/6-31+G(d,p)+LanL2DZ(Pd).[a]

Structure	E_{elec} (Hartree)[b]	E_{elec}+ZPE (Hartree)[c]	H (Hartree)[d]	S (cal mol^{-1} K^{-1})[e]	G (Hartree)[f]	Lowest freq. (cm^{-1})[e]	# imag. freq.
H_2O	–76.4409847	–76.4194817	–76.4157017	45.1	–76.4371277	1601.9	0
$H_2PO_4^-$	–643.764476	–643.727171	–643.720612	74.6	–643.756034	153.7	0
HPO_4^{2-}	–643.276662	–643.251186	–643.245144	72.9	–643.279776	126.4	0
**A**	–2431.21058	–2430.8857	–2430.85854	176.9	–2430.93789	22.9	0
**TS1**	–2507.63498	–2507.28409	–2507.25533	182.8	–2507.33763	–428.5	1
**B**	–2507.6687	–2507.31276	–2507.28464	178.0	–2507.36625	37.5	0
**C**	–2507.23239	–2506.89	–2506.86183	180.8	–2506.94341	27.3	0
**D**	–2506.72803	–2506.39927	–2506.37147	177.8	–2506.45281	32.0	0
**TS2**	–2506.71429	–2506.38772	–2506.35996	178.0	–2506.44129	–450.8	1
**E**	–806.432731	–806.163154	–806.147233	119.2	–806.203186	62.2	0
**F**	–1700.30349	–1700.24903	–1700.23657	111.1	–1700.28816	52.5	0

[a] 1 Hartree = 627.51 kcal mol^{-1}. Thermal corrections at 298.15 K.
[b] Calculated with PCM(H_2O)/ωB97x-D/6-311+G(2d,p)+LanL2DZ(Pd).
[c] Calculated as E_{elec}[PCM(H_2O)/ωB97x-D/6-311+G(2d,p)+LanL2DZ(Pd)] + ZPE[PCM(H_2O)/M06-2X/6-31+G(d,p)+LanL2DZ(Pd)], where ZPE is the zero-point energy obtained at the geometry optimization level.
[d] Calculated as E_{elec}[PCM(H_2O)/ωB97x-D/6-311+G(2d,p)+LanL2DZ(Pd)] + H_{corr}[PCM(H_2O)/M06-2X/6-31+G(d,p)+LanL2DZ(Pd)], where H_{corr} is the thermal correction to enthalpy obtained at the geometry optimization level.
[e] Calculated at the geometry optimization level PCM(H_2O)/M06-2X/6-31+G(d,p)+LanL2DZ(Pd).
[f] Calculated as E_{elec}[PCM(H_2O)/ωB97x-D/6-311+G(2d,p)+LanL2DZ(Pd)] + $G_{corr-QH}$[PCM(H_2O)/M06-2X/6-31+G(d,p)+LanL2DZ(Pd)], where $G_{corr-QH}$ is the thermal correction to Gibbs free energy obtained at the geometry optimization level using quasiharmonic approximations to entropy.

Table S3. Cartesian coordinates of the lowest energy structures calculated with PCM(H_2O)/M06-2X/6-31+G(d,p)/LanL2DZ(Pd).

H_2O

O	0.000000	-0.000000	0.117026
H	0.000000	0.765701	-0.468104
H	-0.000000	-0.765701	-0.468104

$H_2PO_4^-$

P	0.001448	0.095271	0.123336
O	1.276997	-0.386691	-0.792497
H	1.500262	-1.311632	-0.624678
O	-1.287537	-0.343121	-0.795785
H	-1.545211	-1.258462	-0.624753
O	-0.014353	-0.721547	1.388129
O	0.027797	1.593988	0.125076

HPO_4^{2-}

P	-0.135175	-0.031722	0.000849
O	1.459428	0.544615	-0.018120
H	2.067297	-0.204651	-0.002802
O	-0.268237	-0.886836	-1.269736
O	-0.251516	-0.842913	1.301615
O	-0.944634	1.270196	-0.015000

A

C	-1.337427	0.763570	3.153259
C	-1.018842	1.697700	2.170403
C	0.056108	1.476982	1.309727
C	0.816449	0.308166	1.434198
C	0.492271	-0.630477	2.421346
C	-0.580005	-0.402828	3.276773
C	0.362200	2.483761	0.228617
C	1.958965	0.080419	0.528458
C	2.358861	0.983249	-0.420292
C	1.700978	2.260373	-0.487707
C	3.489950	0.685571	-1.363349
H	4.399642	1.225334	-1.072892
C	3.725423	-0.821492	-1.385975
C	3.808296	-1.387758	0.030383
H	-2.174870	0.944035	3.819483
H	-1.603906	2.607451	2.070892
H	1.082646	-1.535313	2.505434
H	-0.829063	-1.136414	4.036531
H	2.892671	-1.315241	-1.901119
O	2.129699	3.207053	-1.151866
O	0.353981	3.778712	0.793280
H	0.839573	4.348928	0.174417
C	-0.741203	2.462713	-0.870527
H	-1.708631	2.650038	-0.394512
H	-0.527980	3.289013	-1.559220
O	2.576704	-1.075242	0.747835
H	4.647240	-1.071907	-1.918940
H	3.224958	1.047969	-2.361929
C	4.959114	-0.790165	0.835007
H	4.998317	-1.246717	1.827483
H	5.907010	-0.980469	0.323203
H	4.840599	0.290033	0.956448
C	3.865196	-2.906137	0.017696
H	3.850128	-3.296156	1.039209
H	3.009507	-3.311584	-0.529530
H	4.786922	-3.236865	-0.468713
C	-0.830885	1.243683	-1.676472
C	-0.894386	0.339546	-2.495536
H	-0.847666	-0.362086	-3.303838
Cl	-0.149879	-2.229409	-0.914714
Cl	-3.123408	-2.214852	0.689877
Pd	-1.958384	-0.690096	-0.708823
Cl	-3.804999	0.824787	-0.597902

TS1

C	-1.338743	-1.027057	3.298746
C	-1.084074	0.259674	2.830890
C	-0.028051	0.495695	1.951295
C	0.792933	-0.566662	1.556399
C	0.533495	-1.860987	2.024840
C	-0.533688	-2.090347	2.886117
C	0.180453	1.884216	1.398164
C	1.931141	-0.311991	0.655649
C	2.235062	0.927609	0.149489
C	1.436947	2.047773	0.532161
C	3.346659	1.121837	-0.844011
H	4.208280	1.612998	-0.375296
C	3.734683	-0.229006	-1.437773
C	3.896311	-1.286051	-0.348244
H	-2.167335	-1.201119	3.977552
H	-1.713246	1.087625	3.144255
H	1.168751	-2.678699	1.704833
H	-0.736215	-3.096455	3.238231
H	2.948682	-0.566778	-2.122619
O	1.680240	3.206365	0.148517
O	0.256812	2.788500	2.486579
H	0.555711	3.638731	2.127060
C	-1.022676	2.300537	0.508988
H	-1.941201	2.166551	1.083261
H	-0.900145	3.365077	0.280938
O	2.642492	-1.399165	0.394834
H	4.669699	-0.159267	-2.001086
H	3.001398	1.798278	-1.633190
C	4.984478	-0.932948	0.661272
H	5.078657	-1.728899	1.404583
H	5.942152	-0.819177	0.145247
H	4.757760	0.001660	1.181641
C	4.111871	-2.668221	-0.940483
H	4.144193	-3.421513	-0.148585
H	3.302903	-2.915607	-1.633444
H	5.060559	-2.691274	-1.483374
C	-0.754110	1.730794	-1.968226
Cl	0.023387	-1.322668	-1.939006
Cl	-2.890524	-2.644497	-0.646445
Pd	-1.917266	-0.401826	-0.848271

Cl	-3.914747	0.576666	0.098924
H	-0.616622	1.459893	-2.996492
O	0.217887	3.465959	-2.248904
H	-0.397077	4.213177	-2.264950
C	-1.098877	1.535527	-0.768216
H	0.797035	3.581620	-1.467669

B

C	-1.509740	-0.020792	3.202351
C	-1.118543	1.095469	2.466123
C	0.025031	1.035918	1.673043
C	0.767810	-0.149802	1.608921
C	0.366820	-1.272261	2.344604
C	-0.769077	-1.204705	3.139711
C	0.388392	2.188764	0.779954
C	1.968843	-0.194607	0.772042
C	2.521312	0.948419	0.162482
C	1.804699	2.115656	0.279929
C	3.874105	0.914964	-0.495628
H	4.440260	1.803070	-0.202645
C	4.615999	-0.351716	-0.080672
C	3.710099	-1.574653	-0.175409
H	-2.399708	0.029348	3.821201
H	-1.703154	2.009955	2.497805
H	0.941703	-2.188271	2.275244
H	-1.084231	-2.073621	3.707110
H	5.491108	-0.515653	-0.714938
O	2.330604	3.242919	-0.139206
O	0.177021	3.461538	1.365953
H	0.537721	3.474893	2.265015
C	-0.551337	2.181858	-0.481355
H	-1.527004	2.465886	-0.081185
H	-0.201573	2.989859	-1.137479
O	2.549163	-1.356508	0.711858
H	4.967309	-0.260392	0.953413
H	3.748715	0.964143	-1.583581
C	4.372867	-2.819697	0.383648
H	3.668015	-3.654961	0.389701
H	5.225802	-3.086948	-0.245293
H	4.728807	-2.643875	1.402049
C	3.156885	-1.802778	-1.576242
H	2.533444	-2.698828	-1.594031
H	2.538683	-0.961349	-1.902363
H	3.989922	-1.923891	-2.274606
C	0.188769	0.651772	-2.262377
Cl	-0.416460	-2.268203	-0.953974
Cl	-3.816051	-2.084349	0.214689
Pd	-2.012398	-0.416965	-0.693999
Cl	-3.743805	1.372051	-0.855135
H	0.169817	-0.263356	-2.845454
O	1.158223	1.567718	-2.612320
H	1.584848	1.293585	-3.432559
C	-0.636789	0.888527	-1.238481
H	1.740238	3.981946	0.105517

C

C	-1.250180	-1.290696	3.166026
C	-1.060354	0.025996	2.754232
C	-0.010001	0.357557	1.897720
C	0.873027	-0.642143	1.474060
C	0.678448	-1.967532	1.884573
C	-0.383788	-2.291297	2.720950
C	0.132317	1.780724	1.414424
C	2.009482	-0.289599	0.605042
C	2.262503	0.982848	0.168336
C	1.389042	2.044890	0.575237
C	3.374527	1.278190	-0.799293
H	4.207723	1.786334	-0.297874
C	3.832909	-0.022751	-1.451370
C	4.032209	-1.121073	-0.409468
H	-2.075752	-1.537023	3.826163
H	-1.738252	0.805018	3.090427
H	1.362030	-2.735290	1.541267
H	-0.534700	-3.320758	3.029152
H	3.069522	-0.364653	-2.159789
O	1.583065	3.225742	0.245059
O	0.182712	2.621188	2.562992
H	0.393188	3.512430	2.244254
C	-1.079452	2.189533	0.541376
H	-1.985329	2.007235	1.124993
H	-1.000844	3.276571	0.394955
O	2.777211	-1.333393	0.303225
H	4.768812	0.116323	-2.000548
H	3.003066	1.975527	-1.557554
C	5.087974	-0.760679	0.632027
H	5.216412	-1.586594	1.336656
H	6.044756	-0.568975	0.137640
H	4.803297	0.132820	1.194448
C	4.328750	-2.460574	-1.062431
H	4.389206	-3.247953	-0.305963
H	3.542421	-2.716193	-1.778403
H	5.284480	-2.410195	-1.591031
C	-0.773144	2.068206	-1.935116
Cl	0.028377	-1.177591	-2.049901
Cl	-2.931227	-2.751387	-0.678665
Pd	-1.926540	-0.321934	-0.804535
Cl	-4.030957	0.611857	0.154880
H	-0.869035	1.578430	-2.898927
O	-0.204802	3.320788	-2.063707
H	0.260141	3.558560	-1.244645
C	-1.169448	1.495697	-0.793742

D

C	-1.341686	0.187083	3.263677
C	-0.991675	1.249940	2.431470
C	0.131835	1.161165	1.610395
C	0.904390	-0.008497	1.616843
C	0.545118	-1.077272	2.446819
C	-0.575528	-0.980177	3.265920
C	0.444594	2.239528	0.617506
C	2.108335	-0.074309	0.768983
C	2.625461	1.005734	0.115817
C	1.891090	2.246384	0.153713
C	3.957331	0.955033	-0.574171
H	4.527554	1.855121	-0.324214
C	4.714874	-0.297706	-0.143268

C	3.792952	-1.516884	-0.156196
H	-2.217703	0.264512	3.900364
H	-1.592044	2.154564	2.410255
H	1.143229	-1.981636	2.436996
H	-0.852697	-1.813384	3.903892
H	5.569292	-0.490863	-0.799276
O	2.365539	3.326279	-0.223066
O	0.135832	3.513593	1.157458
H	0.727280	4.132882	0.698252
C	-0.467555	2.033500	-0.664615
H	-1.449464	2.363805	-0.314999
H	-0.102816	2.773217	-1.394800
O	2.697148	-1.281718	0.774160
H	5.100808	-0.172309	0.875928
H	3.794184	0.971114	-1.659209
C	4.491840	-2.754093	0.381825
H	3.789287	-3.590199	0.441847
H	5.310881	-3.035128	-0.285918
H	4.900723	-2.563390	1.378211
C	3.192871	-1.765111	-1.537345
H	2.568503	-2.663229	-1.516159
H	2.580728	-0.916807	-1.862235
H	3.999290	-1.916117	-2.262568
C	0.314268	0.297538	-2.297214
Cl	-0.827545	-2.623206	-0.903195
Cl	-4.073268	-1.743464	0.652325
Pd	-2.050071	-0.415946	-0.667817
Cl	-3.736897	1.456636	-1.149746
H	0.142477	-0.703389	-2.732411
O	1.311018	0.989029	-2.748200
C	-0.550434	0.691122	-1.307949

TS2

C	-1.448756	0.918314	3.113537
C	-0.935332	1.857024	2.234471
C	0.204335	1.562242	1.459174
C	0.813992	0.290952	1.581582
C	0.277722	-0.650651	2.481794
C	-0.838946	-0.343623	3.240854
C	0.699022	2.459457	0.452368
C	2.016303	0.018681	0.812984
C	2.620784	0.963595	0.023098
C	2.011714	2.262837	-0.116616
C	3.949650	0.705842	-0.627573
H	4.569438	1.603979	-0.544161
C	4.632166	-0.485139	0.038688
C	3.654371	-1.648686	0.205705
H	-2.327618	1.156423	3.705772
H	-1.407374	2.828694	2.123465
H	0.750283	-1.623491	2.566459
H	-1.249262	-1.078554	3.926351
H	5.495372	-0.826092	-0.541935
O	2.548248	3.221273	-0.747190
O	0.300772	3.784438	0.519629
H	0.956655	4.253650	-0.028274
C	-0.396652	1.863142	-1.156140
H	-1.235079	2.400176	-0.725549
H	0.217939	2.448585	-1.845178
O	2.539505	-1.217741	1.025499
H	4.994545	-0.198190	1.033875
H	3.803525	0.527195	-1.701104
C	4.283861	-2.793312	0.984559
H	3.546519	-3.581948	1.161250
H	5.116893	-3.216817	0.416350
H	4.660108	-2.440093	1.949102
C	3.109593	-2.129861	-1.136612
H	2.438248	-2.979363	-0.983062
H	2.552773	-1.336043	-1.643496
H	3.937881	-2.444439	-1.780010
C	0.331836	-0.095954	-2.408772
Cl	-0.839190	-2.713537	-0.778411
Cl	-3.962116	-1.771013	0.795594
Pd	-2.030152	-0.507353	-0.635008
Cl	-3.657980	1.385762	-0.976875
H	0.118120	-1.139988	-2.690189
O	1.319438	0.473342	-2.938068
C	-0.553635	0.507384	-1.475346

E

C	3.437041	-2.046770	0.025363
C	3.321354	-0.677457	0.048633
C	2.040298	-0.046944	-0.003898
C	0.874893	-0.881157	-0.084516
C	1.032651	-2.289719	-0.101978
C	2.281862	-2.869090	-0.049008
C	1.879272	1.343706	0.027091
C	-0.402818	-0.250981	-0.145942
C	-0.540268	1.122776	-0.128215
C	0.622003	1.971302	-0.023705
C	-1.894378	1.771549	-0.222333
H	-1.841875	2.613370	-0.920734
C	-2.949465	0.763140	-0.668158
C	-2.779715	-0.570730	0.062256
H	4.420954	-2.505940	0.065412
H	4.205566	-0.049920	0.107595
H	0.142482	-2.908457	-0.158021
H	2.385145	-3.949581	-0.062929
H	-3.960830	1.145888	-0.495698
O	0.551487	3.264281	0.015530
O	2.976391	2.176527	0.115650
H	2.572277	3.065175	0.119545
H	-2.166953	2.210909	0.747553
H	-2.848848	0.568893	-1.743181
O	-1.476757	-1.106428	-0.245804
C	-2.904500	-0.418004	1.578628
H	-3.872355	0.023667	1.835442
H	-2.828987	-1.398068	2.058561
H	-2.114411	0.223448	1.978408
C	-3.763527	-1.607863	-0.457368
H	-3.577567	-2.577333	0.014234
H	-4.788202	-1.300569	-0.229283
H	-3.663580	-1.719065	-1.541014

F

C	2.221590	-0.001074	-1.650376
H	1.632238	-0.000790	-2.562831

H	3.306331	-0.001764	-1.765878	Cl	-0.141404	-2.458625	-0.066208
C	2.566771	-0.001250	0.732478	H	3.647604	-0.001874	0.479523
Cl	-0.138714	2.459097	-0.066129	O	2.218254	-0.001047	1.899787
Cl	-2.841744	0.000733	0.249818	C	1.662881	-0.000708	-0.434106
Pd	-0.260174	0.000228	-0.113788				

8. Prodrug stability in cell medium

The stability of the prodrug in cell medium was observed by incubating Propargyl-Lap (500 µM) at 37 °C in McCoy's medium and RPMI medium for 24 h. Stability of Propagyl-Lap (11.8 min) was monitored over 24 h with acetophone used as an internal standard (7.2 min). The UV profile of Lapachone (11.4 min), with the internal standard, is plotted as a comparison. The degradation of the prodrug in the cell medium was not observed in 24 h, demonstrating the stability of the prodrug in a complex environment, in the absence of palladium. Acetophenone was used as an internal standard (7.2 min).

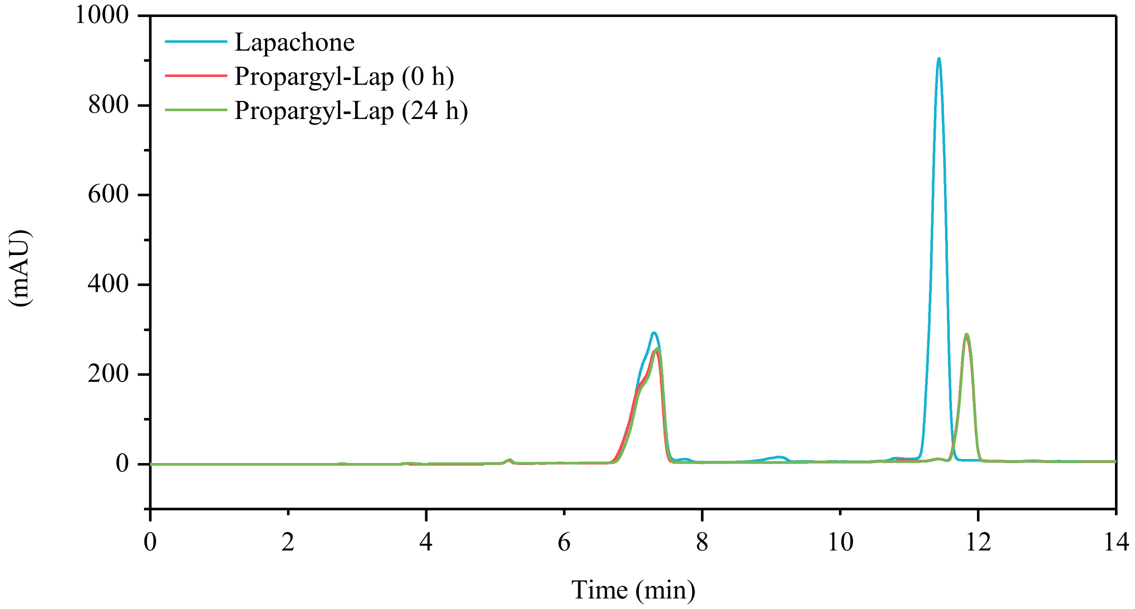

Figure S34. Propargyl-Lap stability in McCoy's medium - (SKBR3).

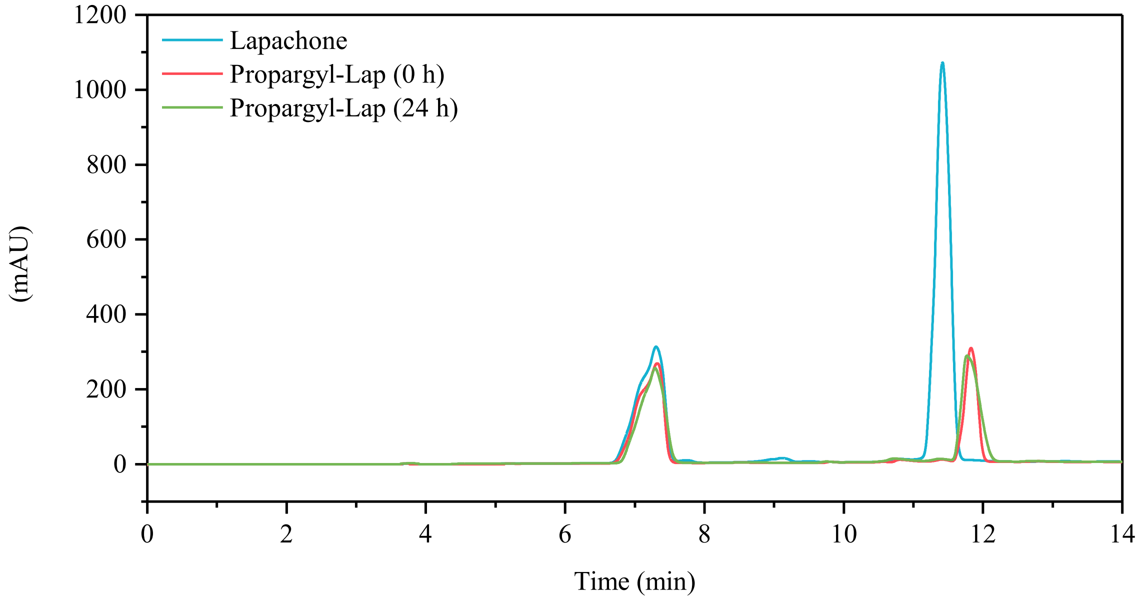

Figure S35. Propargyl-Lap (**2**) stability in RPMI medium - (MOLM13).

9. Cell viability assays

Cells were incubated in a humidified 10% CO_2 – 90% air atmosphere at 37 °C. SKBR-3 cells were cultured in McCoy's 5A (modified), GlutaMAX™ medium supplemented with 10% heat inactivated FBS. SKBR-3 cells (adherent cell line) were passaged by addition of Trypsin-EDTA (0.25%) (Gibco™) every 2–3 days. MOLM-13 cells (suspension cell line) were cultured in RPMI 1640 medium (Gibco™) with 10% heat inactivated FBS and were kept at a density of 1×10^6 cells/mL. Cells were grown to 70% confluency before seeding in 200 µL at 5000 cells/well into a Corning Costar 96-well clear, flat bottom plate for SKBR-3 cell line and round bottom plate for MOLM-13. CellTiter-Blue® assays were employed to determine toxicity and they were performed after replacing the medium with 100 µL of fresh medium followed by the addition of 20 µL of CellTiter-Blue® solution to each well and incubating for 6 h. After this period, the fluorometric determination of cell viability was conducted according to the supplier's guidelines. The fluorescence intensity was measured at λ_{ex} = 555 nm and λ_{em} = 585 nm. The residual fluorescence of CellTiter-Blue® in the cell media was treated as background and therefore it was subtracted from each well. To the cell viability calculation, it was considered the resulting fluorescence intensity in the wells as a percentage of the control, which contained 0.25% of DMSO and was set as 100% viable. All experiments were performed in triplicate.

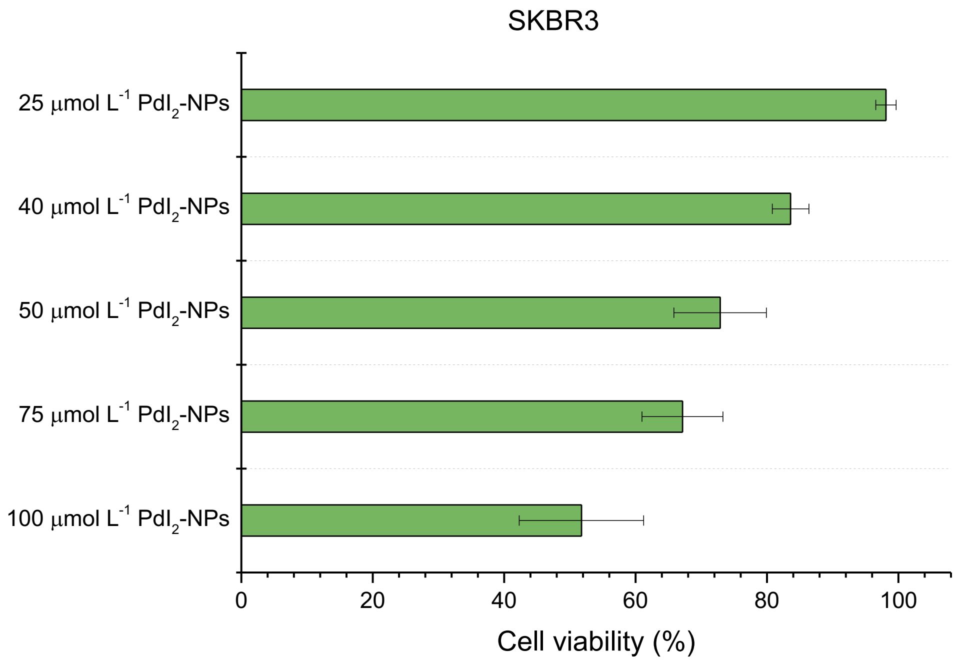

Figure S36. Cell viability of SKBR3 cells after treatment with PdI_2-NPs in different concentrations, after 72 h and 48 h, respectively. Cell viability was determined by CellTiter-Blue® assay. Each experiment was performed in technical triplicates. Error bars represent standard error of the mean (SEM).

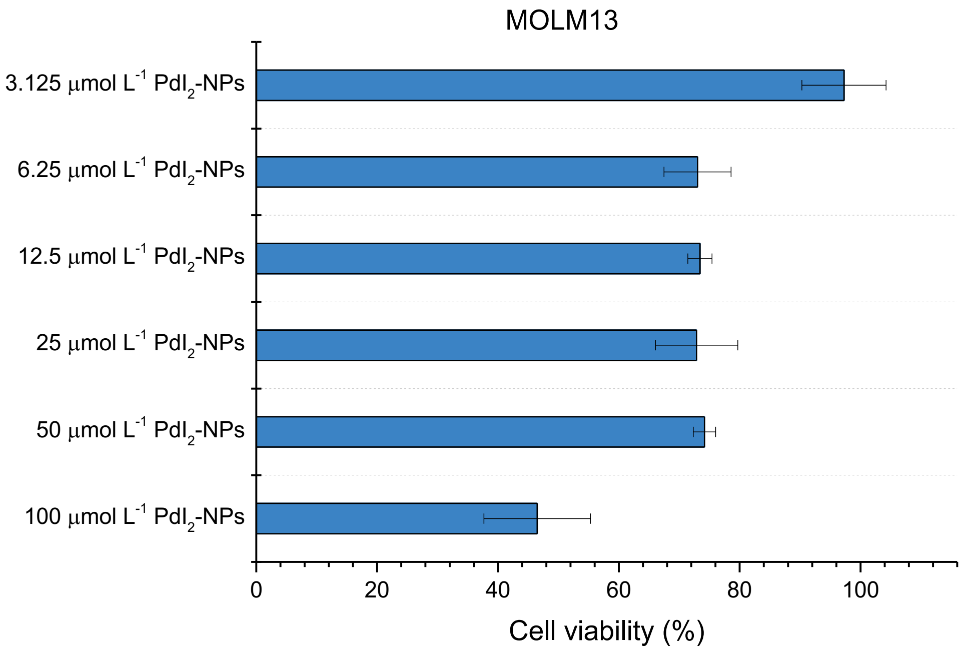

Figure S37. Cell viability of MOLM13 cells after treatment with PdI_2-NPs in different concentrations, after 72 h and 48 h, respectively. Cell viability was determined by CellTiter-Blue® assay. Each experiment was performed in technical triplicates. Error bars represent standard error of the mean (SEM).

10. Propargyl-Lap (2) C–C Decaging in Cancer Cells: Na_2PdCl_4

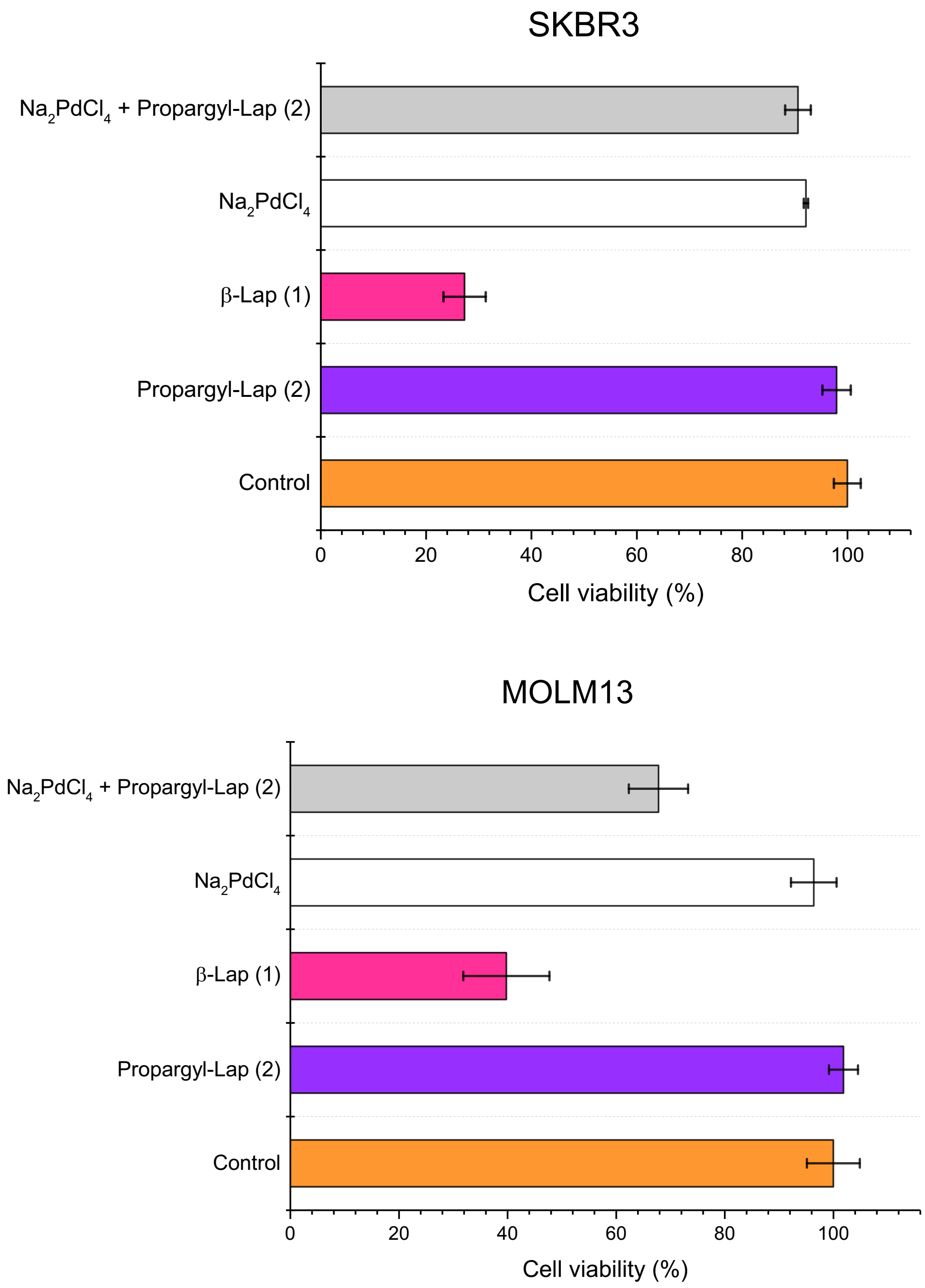

Figure S38. Cell viability of SKBR3 and MOLM13 cells after treatment with Propargyl-Lap (**2**) and subsequent decaging efficiency upon treatment with Na_2PdCl_4, after 72 h and 48 h, respectively. Cell viability was determined by CellTiter-Blue® assay. Each experiment was performed in technical triplicates. Error bars represent standard error of the mean (SEM). [Propargyl-Lap] = 2 μM, [Na_2PdCl_4] = 15 μM, [Lapachone] = 2 μM.

11. Maximum Tolerated Concentration assay in zebrafish larvae

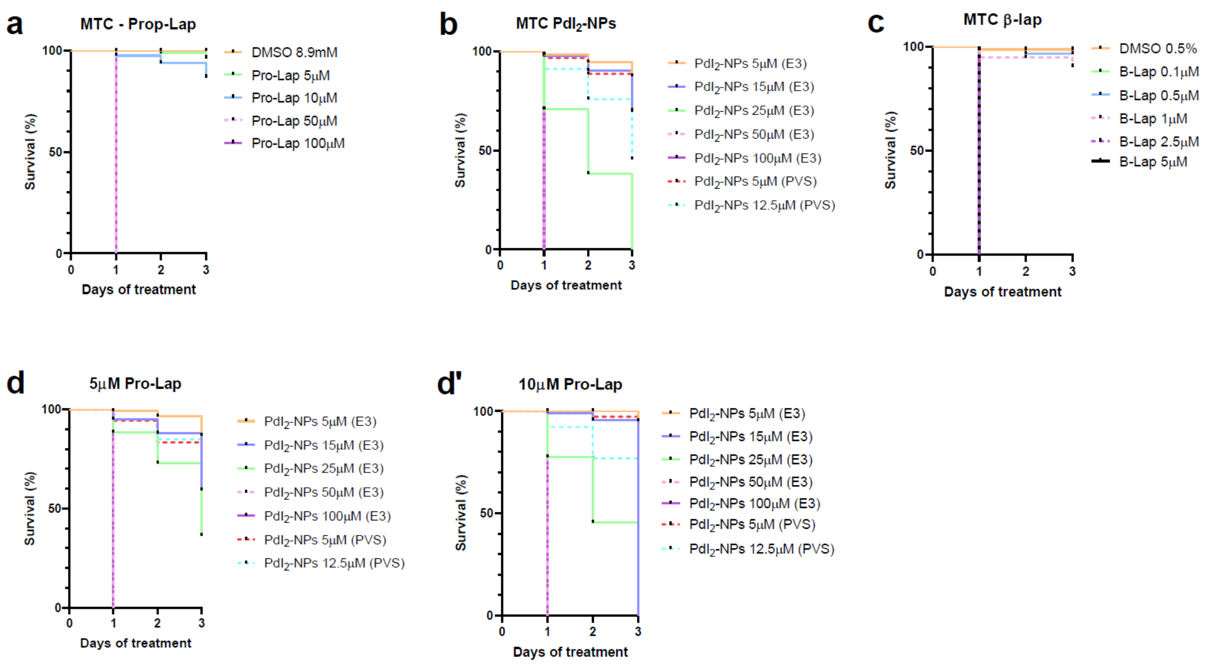

Figure S39. Maximum Tolerated Concentration (MTC) assay. Starting at 3 dpf (days post fertilization), different groups of 30-40 zebrafish larvae were exposed to different concentrations of the compounds, both in single treatment an in combinations. Two different delivery methods were tested for the PdI2-NPs: dilution in the E3 medium or direct injection into the PVS. E3/drug was renewed every day. Propargyl-Lap alone, diluted in the E3 medium (**a**); PdI2-NPs either diluted in E3 or injected into the PVs, always with 8.9mM DMSO in the fish medium (**b**); β-lapachone alone, diluted in the E3 medium (**c**); combination of Propargyl-Lap at 5uM with PdI2-NPs (**c**); combination of Propargyl-Lap at 10uM with PdI2-NPs (**c'**). PdI2-NPs - palladium(II) iodide nanoparticles; Pro-Lap - Propargyl-Lap; β-Lap - β-lapachone.

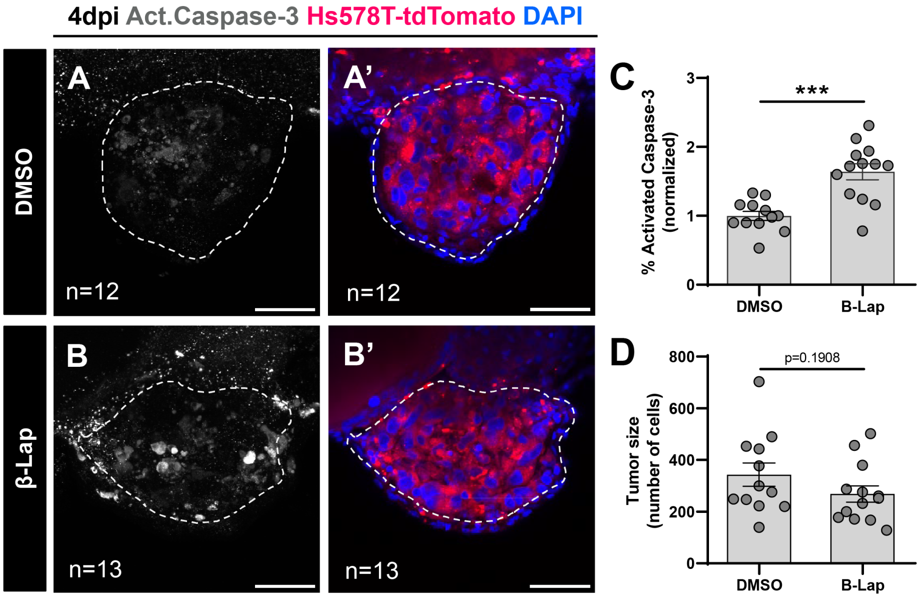

Figure S40. β-lapachone induces apoptosis in zebrafish xenografts. Hs578T-tdTomato TNBC cells were injected into the PVS of 2 days post fertilization zebrafish embryos. At 24hpi, xenografts were randomly distributed into 2 treatment groups: DMSO (control) and β-lapachone at 1uM, with daily E3/drug renewal. At 4 dpi, Hs578T xenografts were fixed, subjected to immunofluorescence, and later imaged by confocal microscopy (DAPI in blue, Hs578T-tdTomato in red, activated caspase-3 in white). Apoptosis (activated caspase 3, fold induction normalized to DMSO controls, **C**: DMSO, **A** vs β-Lap, **B** ***P=0.0001) and tumor size (n° of tumor cells, **D**: DMSO, **A'** vs β-Lap, **B', ns P=0.1908)** were analyzed and quantified. Graphs are presented as average ± SEM. Results are from 1 experiment. The number of xenografts analyzed is indicated in the representative images and each dot in the graphs represents one zebrafish xenograft. Statistical analysis was performed using an unpaired t-test with Welch's correction. Statistical results: ns > 0.05, *P ≤ 0.05, **P ≤ 0.01, ***P ≤ 0.001, and ****P ≤ 0.0001. All images are anterior to the left, posterior to right, dorsal up, and ventral down. Scale bar :50 µm. PdI_2-NPs - palladium (II) iodide nanoparticles; β-Lap - β-lapachone.

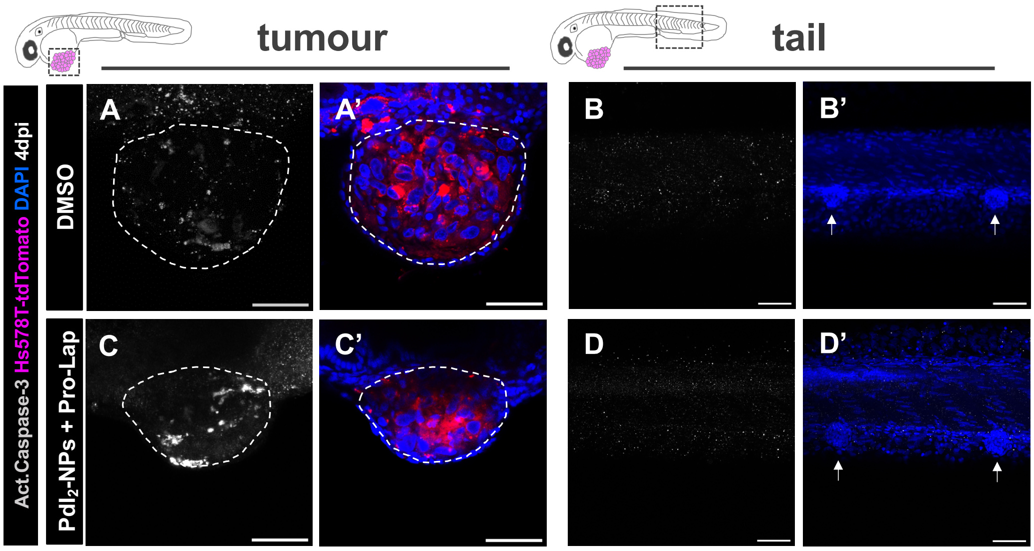

Figure S41. PdI2-NPs-mediated Propargyl-Lap decaging in zebrafish xenografts induce cancer cell death in the tumour but not in non-cancerous organs. Hs578T-tdTomato TNBC cells were injected either alone or together with the PdI2-NPs (5 µM) nanoparticles into the PVS of 2 days post fertilization zebrafish embryos. At 24hpi, xenografts were randomly distributed into 2 treatment groups: DMSO (control) and Propargyl-Lap, with daily E3/drug renewal. At 4 dpi, xenografts were fixed, subjected to immunofluorescence, and later imaged by confocal microscopy (DAPI in blue, activated caspase-3 in white). Apoptosis, assessed by activated caspase 3 staining, was analysed. Images are represented of 5 fish per condition. All images are anterior to the left, posterior to right, dorsal up, and ventral down. Tumors are outlined by a white dashed line and neuromasts are indicated by white arrows. Scale bar: 50 µm. PdI2-NPs - palladium (II) iodide nanoparticles; Prop-Lap - Propargyl-Lap.

12. References

(1) Gaussian 16, Revision C.01, Frisch, M. J.; Trucks, G. W.; Schlegel, H. B.; Scuseria, G. E.; Robb, M. A.; Cheeseman, J. R.; Scalmani, G.; Barone, V.; Petersson, G. A.; Nakatsuji, H.; Li, X.; Caricato, M.; Marenich, A. V.; Bloino, J.; Janesko, B. G.; Gomperts, R.; Mennucci, B.; Hratchian, H. P.; Ortiz, J. V.; Izmaylov, A. F.; Sonnenberg, J. L.; Williams-Young, D.; Ding, F.; Lipparini, F.; Egidi, F.; Goings, J.; Peng, B.; Petrone, A.; Henderson, T.; Ranasinghe, D.; Zakrzewski, V. G.; Gao, J.; Rega, N.; Zheng, G.; Liang, W.; Hada, M.; Ehara, M.; Toyota, K.; Fukuda, R.; Hasegawa, J.; Ishida, M.; Nakajima, T.; Honda, Y.; Kitao, O.; Nakai, H.; Vreven, T.; Throssell, K.; Montgomery, J. A., Jr.; Peralta, J. E.; Ogliaro, F.; Bearpark, M. J.; Heyd, J. J.; Brothers, E. N.; Kudin, K. N.; Staroverov, V. N.; Keith, T. A.; Kobayashi, R.; Normand, J.; Raghavachari, K.; Rendell, A. P.; Burant, J. C.; Iyengar, S. S.; Tomasi, J.; Cossi, M.; Millam, J. M.; Klene, M.; Adamo, C.; Cammi, R.; Ochterski, J. W.; Martin, R. L.; Morokuma, K.; Farkas, O.; Foresman, J. B.; Fox, D. J. Gaussian, Inc., Wallingford CT, 2016.

(2) Zhao, Y.; Truhlar, D. G. The M06 Suite of Density Functionals for Main Group Thermochemistry, Thermochemical Kinetics, Noncovalent Interactions, Excited States, and Transition Elements: Two New Functionals and Systematic Testing of Four M06-Class Functionals and 12 Other Functionals. *Theor. Chem. Acc.* **2008**, *120*, 215–241.

(3) Hay, P. J.; Wadt, W. R. Ab Initio Effective Core Potentials for Molecular Calculations. Potentials for K to Au Including the Outermost Core Orbitals. *J. Chem. Phys.* **1985**, *82*, 299.

(4) Scalmani, G.; Frisch, M. J. Continuous Surface Charge Polarizable Continuum Models of Solvation. I. General Formalism. *J. Chem. Phys.* **2010**, *132* (11), 114110.

(5) Chai, J.-D.; Head-Gordon, M. Long-Range Corrected Hybrid Density Functionals with Damped Atom–Atom Dispersion Corrections. *Phys. Chem. Chem. Phys.* **2008**, *10* (44), 6615.

(6) Ribeiro, R. F.; Marenich, A. V.; Cramer, C. J.; Truhlar, D. G. Use of Solution-Phase Vibrational Frequencies in Continuum Models for the Free Energy of Solvation. *J. Phys. Chem. B* **2011**, *115* (49), 14556–14562.

(7) Hratchian, H. P.; Schlegel, H. B. Following Reaction Pathways Using a Damped Classical Trajectory Algorithm. *J. Phys. Chem. A* **2002**, *106* (1), 165–169.

(8) Póvoa, V.; Rebelo de Almeida, C.; Maia-Gil, M.; Sobral, D.; Domingues, M.; Martinez-Lopez, M.; de Almeida Fuzeta, M.; Silva, C.; Grosso, A. R.; Fior, R. Innate Immune Evasion Revealed in a Colorectal Zebrafish Xenograft Model. *Nat. Commun.* **2021**, *12* (1), 1156.

(9) Calil, F. A.; David, J. S.; Chiappetta, E. R. C.; Fumagalli, F.; Mello, R. B.; Leite, F. H. A.; Castilho, M. S.; Emery, F. S.; Nonato, M. C. Ligand-Based Design, Synthesis and Biochemical Evaluation of Potent and Selective Inhibitors of Schistosoma Mansoni Dihydroorotate Dehydrogenase. *Eur. J. Med. Chem.* **2019**, *167*, 357–366.

(10) Rodrigues, T.; Werner, M.; Roth, J.; da Cruz, E. H. G.; Marques, M. C.; Akkapeddi, P.; Lobo, S. A.; Koeberle, A.; Corzana, F.; da Silva Júnior, E. N.; Werz, O.; Bernardes, G. J. L. Machine Intelligence Decrypts β-Lapachone as an Allosteric 5-Lipoxygenase Inhibitor. *Chem. Sci.* **2018**, *9* (34), 6899–6903.

(11) Inagaki, R.; Ninomiya, M.; Tanaka, K.; Koketsu, M. Synthesis, Characterization, and Antileukemic Properties of Naphthoquinone Derivatives of Lawsone. *ChemMedChem* **2015**, *10* (8), 1413–1423.

(12) Nair, V.; Jayan, C. N.; Ros, S. Novel Reactions of Indium Reagents with 1,2-Diones: A Facile Synthesis of a-Hydroxy Ketones. *Tetrahedron* **2001**, *57* (46), 9453–9459.

(13) Latocheski, E.; Marques, M. V.; Albuquerque, B. L.; Schuh, T. J.; Signori, A. M.; Oliveira, D. C.; Pal, T.; Domingos, J. B. On the Formation of Palladium (II) Iodide Nanoparticles: An In Situ SAXS/XAS Study and Catalytic Evaluation on an Aryl Alkenylation Reaction in Water Medium. *ChemCatChem ChemCatChem* **2019**, *11* (2), 684–688.
